# Noether and partial Noether approach for the nonlinear (3+1)-dimensional elastic wave equations

**DOI:** 10.1371/journal.pone.0315505

**Published:** 2025-01-03

**Authors:** Akhtar Hussain, M. Usman, Fiazuddin Zaman, Ahmed M. Zidan, Jorge Herrera

**Affiliations:** 1 Department of Mathematics and Statistics, The University of Lahore, Lahore, Pakistan; 2 College of Electrical and Mechanical Engineering (CEME), National University of Sciences and Technology (NUST), Islamabad, Pakistan; 3 Department of Mathematics, College of Science, King Khalid University, Abha, Saudi Arabia; 4 Facultad de Ciencias Naturales e Ingenieria, Universidad de Bogota Jorge Tadeo Lozano, Bogota, Colombia; Delft University of Technology, NETHERLANDS, KINGDOM OF THE

## Abstract

The Lie group method is a powerful technique for obtaining analytical solutions for various nonlinear differential equations. This study aimed to explore the behavior of nonlinear elastic wave equations and their underlying physical properties using Lie group invariants. We derived eight-dimensional symmetry algebra for the (3+1)-dimensional nonlinear elastic wave equation, which was used to obtain the optimal system. Group-invariant solutions were obtained using this optimal system. The same analysis was conducted for the damped version of this equation. For the conservation laws, we applied Noether’s theorem to the nonlinear elastic wave equations owing to the availability of a classical Lagrangian. However, for the damped version, we cannot obtain a classical Lagrangian, which makes Noether’s theorem inapplicable. Instead, we used an extended approach based on the concept of a partial Lagrangian to uncover conservation laws. Both techniques account for the conservation laws of linear momentum and energy within the model. These novel approaches add an application of variational calculus to the existing literature. This offers valuable insights and potential avenues for further exploration of the elastic wave equations.

## 1 Introduction

Elasticity [1] is a fundamental concept in many areas of science and engineering such as solid mechanics, material science, and structural engineering. Common examples of elastic media include rubber, metals, springs, and many other materials that can undergo temporary deformation and restore their original shapes when subjected to mechanical loads. In an elastic medium, when stress (force per unit area) is applied, the material undergoes reversible and recoverable deformation, which means that it can return to its initial state after the force is removed. In the case of small deformations, the relationship between the stress and strain (rate of deformation) is linear. However, when the deformations are not small, for example, in the case of hyper-or hypoelasticity, this relationship does not hold.

Elastic wave equations have attracted the interest of numerous researchers owing to their significance in various fields. The Lie group methods have been successfully applied to the study of a variety of elastic wave equations. For instance, Bokhari *et al.* [2] discussed the Lie group’s approach to the elastic wave equation. Mustafa and Masood [3] adopted the same tool over the third-order elastic wave equation with harmonic correction. Usman *et al.* [4, 5] conducted a Lie group analysis of the (1+1) and (2+1) elastic wave equations, adding valuable insights to this domain. In addition, many other authors [6, 7] have also been drawn to explore this area, contributing to the growing body of knowledge in the study of elasticity equations. The application of Lie group methods [8–11] has proven to be a useful approach for understanding the symmetries and properties of these equations, making it an essential tool for researchers in this field.

The linear theory of elasticity posits that the strain tensor $\varkappa_{ij}$ is linearly dependent on the displacement vector *U*_*i*_ according to the equation

$$\begin{array}{c} \varkappa_{ij}=\frac{1}{2}\left(U_{i,j}+U_{j,i}\right) \end{array}$$ (1)

The non-linear elastic model, in contrast, is founded on the concept of non-linear strain, which is represented by

$$\begin{array}{c} \varkappa_{ij}=\frac{1}{2}\left(U_{i,j}+U_{j,i}+U_{k,i}U_{k,j}\right). \end{array}$$ (2)

In the case of a nonlinear strain, the stress is given by the strain rate of a potential function. Among the choices of such a potential functions, the Murnaghan potential appears suitable [1]. This potential is denoted by $W(\varkappa_{ik})$, is expressed as follows

$$\begin{array}{c} W\left(\varkappa_{ik}\right)=\frac{1}{2}\lambda {\left(\varkappa_{mm}\right)}^{2}+\mu {\left(\varkappa_{ik}\right)}^{2}+\frac{1}{3}a_{1}\varkappa_{ik}\varkappa_{im}\varkappa_{km}+a_{2}{\left(\varkappa_{ik}\right)}^{2}\varkappa_{mm}+\frac{1}{3}a_{1}{\left(\varkappa_{mm}\right)}^{3}, \end{array}$$ (3)

where λ and *μ* represent Lame’s coefficients and *a*_1_, *a*_2_ are constants. The stress tensor, denoted by *τ*_*nm*_, can be calculated using the partial derivatives of the Murnaghan potential for the displacement components

$$\begin{array}{c} \tau_{nm}=\frac{\partial W}{\partial U_{m,n}}\cdot \end{array}$$ (4)

The Cauchy equation of motion, in the absence of body forces, is given by

$$\begin{array}{c} \tau_{ij,i}=\rho {\ddot{U}}_{j}. \end{array}$$ (5)

In this context, *U*_*j*_ symbolizes the displacement vector, *τ*_*ij*_ stands for the stress tensor, and *ρ* represents the density of the body.

These equations play a fundamental role in describing the mechanical behavior of elastic materials under the influence of stress and displacement dynamics. The Murnaghan potential represents the elastic energy associated with strain components, while the stress tensor and Cauchy equation of motion help understand the relationship between the stress, displacement, and acceleration of particles within the elastic body.

Considering three-dimensional motion in (*x*_1_, *x*_2_, *x*_3_) and *U*_1_ = *U*, the strain tensor components $\varkappa_{ij}$ for this case are given by

$$\begin{array}{c} \varkappa_{11}=U_{x_{1}}+\frac{1}{2}U_{x_{1}}^{2},\;\varkappa_{22}=\frac{1}{2}U_{x_{2}}^{2},\;\varkappa_{33}=\frac{1}{2}U_{x_{3}}^{2}, \end{array}$$ (6)

$$\begin{array}{c} \varkappa_{12}=\varkappa_{21}=\frac{1}{2}\left(U_{x_{2}}+U_{x_{1}}U_{x_{2}}\right),\;\varkappa_{13}=\varkappa_{31}=\frac{1}{2}\left(U_{x_{3}}+U_{x_{1}}U_{x_{3}}\right),\;\varkappa_{23}=\varkappa_{32}=\frac{1}{2}U_{x_{2}}U_{x_{3}}. \end{array}$$ (7)

For the three-dimensional case, the Murnaghan potential (3) takes the form

$$\begin{array}{c} \begin{array}{cc} W= & \frac{1}{2}\lambda {\left(\varkappa_{11}+\varkappa_{22}+\varkappa_{33}\right)}^{2}+\mu \left(\varkappa_{11}^{2}+\varkappa_{22}^{2}+\varkappa_{33}^{2}+\varkappa_{12}^{2}+\varkappa_{21}^{2}+\varkappa_{13}^{2}+\varkappa_{31}^{2}+\varkappa_{23}^{2}+\varkappa_{32}^{2}\right)\\ & +\frac{1}{3}a_{1}(\varkappa_{11}^{3}+3\varkappa_{11}\varkappa_{12}^{2}+3\varkappa_{11}\varkappa_{13}^{2}+3\varkappa_{33}\varkappa_{13}^{2}+\varkappa_{33}^{3}+3\varkappa_{33}\varkappa_{32}^{2}+6\varkappa_{12}\varkappa_{13}\varkappa_{32}+\varkappa_{22}^{3}\\ & +\varkappa_{22}\varkappa_{12}^{2}+3\varkappa_{22}\varkappa_{32}^{2})+a_{2}(\varkappa_{11}^{2}+\varkappa_{22}^{2}+\varkappa_{33}^{2}+\varkappa_{12}^{2}+\varkappa_{21}^{2}+\varkappa_{13}^{2}+\varkappa_{31}^{2}+\varkappa_{23}^{2}+\\ & \varkappa_{32}^{2})\left(\varkappa_{11}+\varkappa_{22}+\varkappa_{33}\right)+\frac{1}{3}a_{3}{\left(\varkappa_{11}+\varkappa_{22}+\varkappa_{33}\right)}^{3}. \end{array} \end{array}$$ (8)

This leads to the following expression for W

$$\begin{array}{c} W=\left(\frac{\lambda }{2}+\mu \right)U_{x_{1}}^{2}+\left(\frac{\lambda }{2}+\mu +\frac{a_{1}}{3}+a_{1}+\frac{a_{3}}{3}\right)U_{x_{1}}^{3}+\frac{\mu }{2}\left(U_{x_{2}}^{2}+U_{x_{3}}^{2}\right)+\left(\frac{\lambda }{2}+\mu +\frac{a_{1}}{4}+\frac{a_{2}}{2}\right)\left(U_{x_{1}}U_{x_{2}}^{2}+U_{x_{1}}U_{x_{3}}^{2}\right). \end{array}$$ (9)

The stress components can be calculated using the Eq (4) and are given by

$$\begin{array}{c} \begin{array}{c} \tau_{11}=\left(\lambda +2\mu \right)U_{x_{1}}+3\left(\frac{\lambda }{2}+\mu +\frac{a_{1}}{3}+a_{1}+\frac{a_{3}}{3}\right)U_{x_{1}}^{2}+\left(\frac{\lambda }{2}+\mu +\frac{a_{1}}{4}+\frac{a_{2}}{2}\right)\left(U_{x_{2}}^{2}+U_{x_{3}}^{2}\right),\\ \tau_{21}=\mu U_{x_{2}}+\left(\lambda +2\mu +\frac{a_{1}}{2}+a_{1}\right)U_{x_{1}}U_{x_{2}},\\ \tau_{31}=\mu U_{x_{3}}+\left(\lambda +2\mu +\frac{a_{1}}{2}+a_{1}\right)U_{x_{1}}U_{x_{3}}. \end{array} \end{array}$$ (10)

The Cauchy equation of motion (5) takes the following form in the three-dimensional case

$$\begin{array}{c} \tau_{11,1}+\tau_{21,2}+\tau_{31,3}=\rho U_{\tau \tau }, \end{array}$$ (11)

and the components of the potential are given by

$$\begin{array}{c} \begin{array}{cc} \tau_{11,1} & =\left(\lambda +2\mu \right)U_{x_{1}x_{1}}+6\left(\frac{\lambda }{2}+\mu +\frac{a_{1}}{3}+a_{1}+\frac{a_{1}}{3}\right)U_{x_{1}}U_{x_{1}x_{1}}+2(\frac{\lambda }{2}+\mu +\frac{a_{1}}{4}\\ & +\frac{a_{1}}{2})\left(U_{x_{2}}U_{x_{1}x_{2}}+U_{x_{3}}U_{x_{1}x_{3}}\right),\\ \tau_{21,2} & =\mu U_{x_{2}x_{2}}+\left(\lambda +2\mu +\frac{a_{1}}{2}+a_{1}\right)\left(U_{x_{2}}U_{x_{1}x_{2}}+U_{x_{1}}U_{x_{2}x_{2}}\right),\\ \tau_{31,3} & =\mu U_{x_{3}x_{3}}+\left(\lambda +2\mu +\frac{a_{1}}{2}+a_{2}\right)\left(U_{x_{1}}U_{x_{3}x_{3}}+U_{x_{3}}U_{x_{1}x_{3}}\right). \end{array} \end{array}$$ (12)

Lastly, by utilizing Eq (10), we derive the (3+1)-dimensional nonlinear elastic wave equation as follows

$$\begin{array}{c} \begin{array}{cc} & U_{\tau \tau }-AU_{x_{1}x_{1}}-B\left(U_{x_{2}x_{2}}+U_{x_{3}x_{3}}\right)-CU_{x_{1}}U_{x_{1}x_{1}}-D(U_{x_{1}}U_{x_{2}x_{2}}+U_{x_{1}}U_{x_{3}x_{3}}\\ & +2U_{x_{2}}U_{x_{1}x_{2}}+2U_{x_{3}}U_{x_{1}x_{3}})=0. \end{array} \end{array}$$ (13)

In (13), $A=\frac{\lambda +2\mu }{\rho }$, $B=\frac{\mu }{\rho }$, $C=\frac{3\left(\lambda +2\mu \right)+2\left(a_{1}+3a_{2}+a_{3}\right)}{\rho }$, $D=\frac{1}{\rho }\left(\lambda +2\mu +\frac{a_{1}}{2}+a_{2}\right)$.

In Eq (13), λ and *μ* are Lame’s coefficients and *a*_1_, *a*_2_, and *a*_3_ are the Murnaghan constants. These expressions provide essential insights into the behavior of the (3+1)-nonlinear elastic wave equation and the underlying physical properties of the elastic medium under consideration. The aim of this study was to investigate the underlying properties of elastic media. The Lie group method [12–15] was used to study these properties.

This paper is organized into several sections. In Section 2, we conduct a group analysis and establish an optimal system for the (3+1)-nonlinear elastic wave equation. Section 3 explores group-invariant solutions using the identified optimal system. In Section 4, the authors introduce Noether’s approach for formulating conservation laws and present Noether’s symmetry generators. In Section 5, the focus is shifted to the Lie group and the optimal system of the damped version of the considered equation. This paper proceeds to Section 6, in which symmetry reductions are discussed. Section 7 discusses the partial Lagrangian approach employed to investigate conservation laws. A graphical analysis of the obtained results is presented in Section 8. Finally, in Section 9, we conclude the paper and highlight potential future research directions.

## 2 Group analysis of the Eq (13)

This section presents a comprehensive Lie symmetry analysis of (13). For this purpose, we consider the vector field given by [16]

$$\Lambda =\delta_{1}\frac{\partial }{\partial x_{1}}+\delta_{2}\frac{\partial }{\partial x_{2}}+\delta_{3}\frac{\partial }{\partial x_{3}}+\delta_{4}\frac{\partial }{\partial \tau }+\Psi \frac{\partial }{\partial U}\cdot$$

Since the equation under consideration is of second order, we prolong our vector field as follows

$$\begin{array}{c} \Lambda^{\left[2\right]}=\delta_{1}\frac{\partial }{\partial x_{1}}+\delta_{2}\frac{\partial }{\partial x_{2}}+\delta_{3}\frac{\partial }{\partial x_{3}}+\delta_{4}\frac{\partial }{\partial \tau }+\Psi \frac{\partial }{\partial U}+\Psi^{\left[x_{1}\right]}\frac{\partial }{\partial U_{x_{1}}}+\Psi^{\left[x_{2}\right]}\frac{\partial }{\partial U_{x_{2}}}+\Psi^{\left[x_{3}\right]}\frac{\partial }{\partial U_{x_{3}}}+\Psi^{\left[\tau \right]}\frac{\partial }{\partial U_{\tau }}\\ +\Psi^{\left[x_{1}x_{1}\right]}\frac{\partial }{\partial U_{x_{1}x_{1}}}+\Psi^{\left[x_{1}x_{2}\right]}\frac{\partial }{\partial U_{x_{1}x_{2}}}+\Psi^{\left[x_{2}x_{2}\right]}\frac{\partial }{\partial U_{x_{2}x_{2}}}+\Psi^{\left[x_{1}x_{3}\right]}\frac{\partial }{\partial U_{x_{1}x_{3}}}+\Psi^{\left[x_{2}x_{3}\right]}\frac{\partial }{\partial U_{x_{2}x_{3}}}+\Psi^{\left[x_{3}x_{3}\right]}\frac{\partial }{\partial U_{x_{3}x_{3}}}\\ +\Psi^{\left[x_{1}\tau \right]}\frac{\partial }{\partial U_{x_{1}\tau }}+\Psi^{\left[x_{2}\tau \right]}\frac{\partial }{\partial U_{x_{2}\tau }}+\Psi^{\left[x_{3}\tau \right]}\frac{\partial }{\partial U_{x_{3}\tau }}+\Psi^{\left[\tau \tau \right]}\frac{\partial }{\partial U_{\tau \tau }}, \end{array}$$

where, $\Psi^{\left[x_{1}\right]},\Psi^{\left[x_{2}\right]},\Psi^{\left[x_{3}\right]},\Psi^{\left[\tau \right]},\Psi^{\left[x_{1}x_{1}\right]},\Psi^{\left[x_{1}x_{2}\right]},\Psi^{\left[x_{2}x_{2}\right]},\Psi^{\left[x_{1}x_{3}\right]},\Psi^{\left[x_{2}x_{3}\right]},\Psi^{\left[x_{3}x_{3}\right]},\Psi^{\left[x_{2}\tau \right]},\Psi^{\left[x_{2}\tau \right]},\Psi^{\left[x_{3}\tau \right]}$ and $\Psi^{\left[\tau \tau \right]}$ are given as follows

$$\begin{array}{c} \begin{array}{c} \Psi^{\left[x_{1}\right]}=D_{x_{1}}\Psi -U_{x_{1}}D_{x_{1}}\delta_{1}-U_{x_{2}}D_{x_{1}}\delta_{2}-U_{x_{3}}D_{x_{1}}\delta_{3}-U_{\tau }D_{x_{1}}\delta_{4}\\ \Psi^{\left[x_{2}\right]}=D_{x_{2}}\Psi -U_{x_{1}}D_{x_{2}}\delta_{1}-U_{x_{2}}D_{x_{2}}\delta_{2}-U_{x_{3}}D_{x_{2}}\delta_{3}-U_{\tau }D_{x_{2}}\delta_{4}\\ \Psi^{\left[x_{3}\right]}=D_{x_{3}}\Psi -U_{x_{1}}D_{x_{3}}\delta_{1}-U_{x_{2}}D_{\delta_{2}}-U_{x_{3}}D_{x_{3}}\delta_{3}-U_{\tau }D_{x_{3}}\delta_{4}\\ \Psi^{\left[\tau \right]}=D_{\tau }\Psi -U_{x_{1}}D_{\tau }\delta_{1}-U_{x_{2}}D_{\tau }\delta_{2}-U_{x_{3}}D_{\tau }\delta_{3}-U_{\tau }D_{\tau }\delta_{4}\\ \Psi^{\left[x_{1}x_{1}\right]}=D_{x_{1}}\Psi^{\left[x_{1}\right]}-U_{x_{1}x_{1}}D_{x_{1}}\delta_{1}-U_{x_{1}x_{2}}D_{x_{1}}\delta_{2}-U_{x_{1}x_{3}}D_{x_{1}}\delta_{3}-U_{x_{1}\tau }D_{x_{1}}\delta_{4}\\ \Psi^{\left[x_{2}x_{2}\right]}=D_{x_{2}}\Psi^{\left[x_{2}\right]}-U_{x_{1}x_{2}}D_{x_{2}}\delta_{1}-U_{x_{2}x_{2}}D_{x_{2}}\delta_{2}-U_{x_{2}x_{3}}D_{x_{2}}\delta_{3}-U_{x_{2}\tau }D_{x_{2}}\delta_{4}\\ \Psi^{\left[x_{3}x_{3}\right]}=D_{x_{3}}\Psi^{\left[x_{3}\right]}-U_{x_{1}x_{3}}D_{x_{3}}\delta_{1}-U_{x_{2}x_{3}}D_{x_{3}}\delta_{2}-U_{\tau \tau }D_{x_{3}}\delta_{3}-U_{x_{3}\tau }D_{x_{3}}\delta_{4}\\ \Psi^{\left[\tau \tau \right]}=D_{\tau }\Psi^{\left[\tau \right]}-U_{x_{1}\tau }D_{\tau }\delta_{1}-U_{x_{2}\tau }D_{\tau }\delta_{2}-U_{x_{3}\tau }D_{\tau }\delta_{3}-U_{\tau \tau }D_{\tau }\delta_{4}\\ \Psi^{\left[x_{1}x_{2}\right]}=D_{x_{2}}\Psi^{\left[x_{1}\right]}-U_{x_{1}x_{1}}D_{x_{2}}\delta_{1}-U_{x_{1}x_{2}}D_{x_{2}}\delta_{2}-U_{x_{1}x_{3}}D_{x_{2}}\delta_{3}-U_{x_{1}\tau }D_{x_{2}}\delta_{4}\\ \Psi^{\left[x_{1}\tau \right]}=D_{\tau }\Psi^{\left[x_{1}\right]}-U_{x_{1}x_{1}}D_{\tau }\delta_{1}-U_{x_{1}x_{2}}D_{\tau }\delta_{2}-U_{x_{1}x_{3}}D_{\tau }\delta_{3}-U_{x_{1}\tau }D_{\tau }\delta_{4}\\ \Psi^{\left[x_{2}\tau \right]}=D_{\tau }\Psi^{\left[x_{2}\right]}-U_{x_{1}x_{2}}D_{\tau }\delta_{1}-U_{x_{2}x_{2}}D_{\tau }\delta_{2}-U_{x_{2}x_{3}}D_{\tau }\delta_{3}-U_{x_{2}\tau }D_{\tau }\delta_{4}\\ \Psi^{\left[x_{1}x_{3}\right]}=D_{x_{3}}\Psi^{\left[x_{1}\right]}-U_{x_{1}x_{1}}D_{x_{3}}\delta_{1}-U_{x_{1}x_{2}}D_{x_{3}}\delta_{2}-U_{x_{1}x_{3}}D_{x_{3}}\delta_{3}-U_{x_{1}\tau }D_{x_{3}}\delta_{4}\\ \Psi^{\left[x_{2}x_{3}\right]}=D_{x_{3}}\Psi^{\left[x_{2}\right]}-U_{x_{1}x_{2}}D_{x_{3}}\delta_{1}-U_{x_{2}x_{2}}D_{x_{3}}\delta_{2}-U_{x_{2}x_{3}}D_{x_{3}}\delta_{3}-U_{x_{2}\tau }D_{x_{3}}\delta_{4}\\ \Psi^{\left[x_{3}\tau \right]}=D_{\tau }\Psi^{\left[x_{3}\right]}-U_{x_{1}x_{3}}D_{\tau }\delta_{1}-U_{x_{2}x_{3}}D_{\tau }\delta_{2}-U_{\tau \tau }D_{\tau }\delta_{3}-U_{nu_{3}\tau }D_{\tau }\delta_{4}, \end{array} \end{array}$$ (14)

where **D**_*α*_ denotes the total derivative of *α*.

The invariance criterion of PDE is

$$\begin{array}{c} \begin{array}{cc} & \Lambda^{\left[2\right]}(U_{\tau \tau }-AU_{x_{1}x_{1}}-B\left(U_{x_{2}x_{2}}+U_{x_{3}x_{3}}\right)-CU_{x_{1}}U_{x_{1}x_{1}}-D\left(U_{x_{1}}U_{x_{2}x_{2}}+U_{x_{1}}U_{x_{3}x_{3}}\right)\\ & -2D\left(U_{x_{2}}U_{x_{1}x_{2}}+U_{x_{3}}U_{x_{1}x_{3}}\right){)|}_{\left(13\right)}=0. \end{array} \end{array}$$ (15)

The invariance condition (15) which lead to the following after some calculations. After performing some calculations we get,

$$\begin{array}{c} \begin{array}{cc} & \delta_{1_{x_{1}}}=\delta_{2_{x_{2}}}=\delta_{3_{x_{3}}}=\delta_{4_{\tau }}=\Psi_{U},\\ & \delta_{1_{x_{2}}}=\delta_{1_{x_{3}}}=\delta_{1_{\tau }}=\delta_{1_{U}}=0,\\ & \delta_{4_{x_{1}}}=\delta_{4_{x_{2}}}=\delta_{4_{x_{3}}}=\delta_{4_{U}}=0,\\ & \delta_{2_{x_{1}}}=\delta_{2_{\tau }}=\delta_{2_{U}}=0,\\ & \delta_{3_{x_{1}}}=\delta_{3_{\tau }}=\delta_{3_{U}}=0,\\ & \Psi_{x_{1}}=\Psi_{x_{2}}=\Psi_{x_{3}}=0,\\ & \delta_{4_{\tau \tau }}=0,\\ & \delta_{2_{x_{3}x_{3}}}=0,\\ & \Psi_{\tau \tau }=0,\\ & \delta_{3_{x_{2}}}+\delta_{2_{x_{3}}}=0. \end{array} \end{array}$$ (16)

So the infinitesimals are

$$\begin{array}{c} \begin{array}{cc} & \delta_{1}=C_{1}x_{1}+C_{2},\\ & \delta_{2}=C_{1}x_{2}+C_{3}x_{3}+C_{4},\\ & \delta_{3}=C_{1}x_{3}-C_{3}x_{2}+C_{5},\\ & \delta_{4}=C_{1}\tau +C_{6},\\ & \Psi =C_{1}U+C_{7}\tau +C_{8}. \end{array} \end{array}$$ (17)

We obtain the following symmetry algebra

$$\Lambda_{1}=\frac{\partial }{\partial \tau },\;\Lambda_{2}=\frac{\partial }{\partial U},\;\Lambda_{3}=\frac{\partial }{\partial x_{1}},\;\Lambda_{4}=\frac{\partial }{\partial x_{2}},$$

$$\Lambda_{5}=\frac{\partial }{\partial x_{3}},\;\Lambda_{6}=\tau \frac{\partial }{\partial U},\;\Lambda_{7}=x_{3}\frac{\partial }{\partial x_{2}}-x_{2}\frac{\partial }{\partial x_{3}},$$

$$\Lambda_{8}=x_{1}\frac{\partial }{\partial x_{1}}+x_{2}\frac{\partial }{\partial x_{2}}+x_{3}\frac{\partial }{\partial x_{3}}+\tau \frac{\partial }{\partial \tau }+U\frac{\partial }{\partial U}\cdot$$

Table 1 shows the commutator relation for the symmetry algebra (2). The adjoint representations of the symmetry algebra (15) are presented in Tables 2 and 3.

**Table 1:** Commutator table.

[Λ_*i*_, Λ_*j*_]	Λ_1_	Λ_2_	Λ_3_	Λ_4_	Λ_5_	Λ_6_	Λ_7_	Λ_8_
Λ_1_	0	0	0	0	0	Λ_2_	0	Λ_1_
Λ_2_	0	0	0	0	0	0	0	Λ_2_
Λ_3_	0	0	0	0	0	0	0	Λ_3_
Λ_4_	0	0	0	0	0	0	−Λ_5_	Λ_4_
Λ_5_	0	0	0	0	0	0	Λ_4_	Λ_5_
Λ_6_	−Λ_2_	0	0	0	0	0	0	0
Λ_7_	0	0	0	Λ_5_	−Λ_4_	0	0	0
Λ_8_	−Λ_1_	−Λ_2_	−Λ_3_	−Λ_4_	-Λ_5_	0	0	0

**Table 2:** Adjoint table.

*Ad*(*e*^*ϵ*^)	Λ_1_	Λ_2_	Λ_3_	Λ_4_
Λ_1_	Λ_1_	Λ_2_	Λ_3_	Λ_4_
Λ_2_	Λ_1_	Λ_2_	Λ_3_	Λ_4_
Λ_3_	Λ_1_	Λ_2_	Λ_3_	Λ_4_
Λ_4_	Λ_1_	Λ_2_	Λ_3_	Λ_4_
Λ_5_	Λ_1_	Λ_2_	Λ_3_	Λ_4_
Λ_6_	Λ_1_ + *ϵ*Λ_2_	Λ_2_	Λ_3_	Λ_4_
Λ_7_	Λ_1_	Λ_2_	Λ_3_	cos(*ϵ*)Λ_4_ − sin(*ϵ*)Λ_5_
Λ_8_	*e*^*ϵ*^Λ_1_	*e*^*ϵ*^Λ_2_	*e*^*ϵ*^Λ_3_	*e*^*ϵ*^Λ_4_

**Table 3:** Adjoint table.

*Ad*(*e*^*ϵ*^)	Λ_5_	Λ_6_	Λ_7_	Λ_8_
Λ_1_	Λ_5_	Λ_6_ − *ϵ*Λ_2_	Λ_7_	Λ_8_ − *ϵ*Λ_1_
Λ_2_	Λ_5_	Λ_6_	Λ_7_	Λ_8_ − *ϵ*Λ_2_
Λ_3_	Λ_5_	Λ_6_	Λ_7_	Λ_8_ − *ϵ*Λ_3_
Λ_4_	Λ_5_	Λ_6_	Λ_7_ + *ϵ*Λ_5_	Λ_8_ − *ϵ*Λ_4_
Λ_5_	Λ_5_	Λ_6_	Λ_7_ − *ϵ*Λ_4_	Λ_8_ − *ϵ*Λ_5_
Λ_6_	Λ_5_	Λ_6_	Λ_7_	Λ_8_
Λ_7_	sin(*ϵ*)Λ_4_ + cos(*ϵ*)Λ_5_	Λ_6_	Λ_7_	Λ_8_
Λ_8_	*e*^*ϵ*^Λ_5_	Λ_6_	Λ_7_	Λ_8_

### 2.1 Optimal system

The optimal system of subalgebras is a concept in the context of Lie symmetry analysis. This was first introduced by Ibragimov [17] and then carried out by Olver [18]. When analyzing the symmetries of a differential equation using the Lie group method, we often encounter Lie algebra consisting of several infinitesimal generators. However, not all these generators are linearly independent. The optimal system of subalgebras is the maximal set of linearly independent generators that provide a complete description of the symmetries of the differential equation. It represents the minimal set of generators needed to construct all symmetries and invariant solutions of the equation. Here, we discuss the optimal system for (13):

Consider a general element Λ of Lie algebra *L*^8^ given by,

$$\begin{array}{c} \Lambda =\mu_{1}\Lambda_{1}+\mu_{2}\Lambda_{2}+\mu_{3}\Lambda_{3}+\mu_{4}\Lambda_{4}+\mu_{5}\Lambda_{5}+\mu_{6}\Lambda_{6}+\mu_{7}\Lambda_{7}+\mu_{8}\Lambda_{8}, \end{array}$$ (18)

where *μ* denotes arbitrary constants.

**Case 1:** *μ*_8_ ≠ 0, *μ*_7_ ≠ 0. By the adjoint action of Λ_1_, Λ_3_ and Λ_4_, (18) simplifies to Λ = *μ*_6_Λ_6_ + *μ*_7_Λ_7_ + *μ*_8_Λ_8_. Therefore, the corresponding subalgebra is $R_{1}=\Lambda_{6}+\kappa \Lambda_{7}+\sigma \Lambda_{8}$ *κ*, *σ* ≠ 0.

**Case 2:** *μ*_8_ ≠ 0, *μ*_7_ = 0. By the adjoint action of Λ_1_, Λ_3_, Λ_4_ and Λ_5_, (18) simplifies to Λ = *μ*_6_Λ_6_ + *μ*_8_Λ_8_. Hence, the corresponding subalgebra is $R_{2}=\Lambda_{6}+\kappa \Lambda_{8}$ *κ* ≠ 0.

**Case 3:** *μ*_8_ ≠ 0, *μ*_7_ ≠ 0, *μ*_6_ = 0. By the adjoint action of Λ_1_, Λ_2_, Λ_3_ and Λ_4_, (18) simplifies to Λ = *μ*_7_Λ_7_ + *μ*_8_Λ_8_. Hence, the corresponding subalgebra is $R_{3}=\Lambda_{7}+\kappa \Lambda_{8}$ *κ* ≠ 0.

**Case 4:** *μ*_8_ ≠ 0, *μ*_7_ = 0, *μ*_6_ = 0. By the adjoint action of Λ_1_, Λ_2_, Λ_3_, Λ_4_ and Λ_5_, (18) simplifies to Λ = *μ*_8_Λ_8_. Therefore, the corresponding subalgebra is $R_{4}=\Lambda_{8}$.

**Case 5:** *μ*_8_ = 0, *μ*_7_ ≠ 0, *μ*_3_ ≠ 0. From the adjoint action of Λ_1_, Λ_4_, Λ_5_ and Λ_8_, Eq (18) simplifies to Λ = *μ*_1_Λ_1_ + *μ*_3_Λ_3_ + *e*^−*ϵ*^*μ*_6_Λ_6_ + *e*^−*ϵ*^*μ*_7_Λ_7_. Therefore, the corresponding subalgebra is $R_{5}=\Lambda_{1}+\kappa \Lambda_{3}\pm \Lambda_{6}\pm \Lambda_{7}$ *κ* ≠ 0.

**Case 6:** *μ*_8_ = 0, *μ*_7_ ≠ 0, *μ*_6_ = 0, *μ*_3_ ≠ 0. By the adjoint action of Λ_4_, Λ_5_, Λ_6_ and Λ_8_, (18) simplifies to Λ = *μ*_1_Λ_1_ + *μ*_3_Λ_3_ + *e*^−*ϵ*^*μ*_7_Λ_7_. Therefore, the corresponding subalgebra is $R_{6}=\Lambda_{1}+\kappa \Lambda_{3}\pm \Lambda_{7}$, and *κ* ≠ 0.

**Case 7:** *μ*_8_ = 0, *μ*_7_ = 0, *μ*_6_ ≠ 0, *μ*_3_ ≠ 0. By the adjoint action of Λ_6_, Λ_7_ and Λ_8_, (18) simplifies to Λ = *μ*_1_Λ_1_ + *μ*_3_Λ_3_ + *e*^−*ϵ*^*μ*_6_Λ_6_. Therefore, the corresponding subalgebra is $R_{7}=\Lambda_{1}+\kappa \Lambda_{3}\pm \Lambda_{6}$, and *κ* ≠ 0.

**Case 8:** *μ*_8_ = 0, *μ*_7_ = 0, *μ*_6_ = 0, *μ*_3_ ≠ 0. By the adjoint action of Λ_6_ and Λ_7_, (18) simplifies to Λ = *μ*_1_Λ_1_ + *μ*_3_Λ_3_. Therefore, the corresponding subalgebra is $R_{8}=\Lambda_{1}+\kappa \Lambda_{3}$, and *κ* ≠ 0.

**Case 9:** *μ*_8_ = 0, *μ*_7_ = 0, *μ*_6_ = 0, *μ*_5_ = 0, *μ*_4_ ≠ 0, *μ*_3_ ≠ 0. By the adjoint action of Λ_6_, (18) simplifies to Λ = *μ*_1_Λ_1_ + *μ*_3_Λ_3_ + *μ*_4_Λ_4_. Thus, $R_{9}=\Lambda_{1}+\kappa \Lambda_{3}+\sigma \Lambda_{4}$ *κ*, *σ* ≠ 0.

**Case 10:** *μ*_8_ = 0, *μ*_7_ = 0, *μ*_6_ = 0, *μ*_1_ = 0, *μ*_5_ = 0, *μ*_4_ ≠ 0, *μ*_3_ ≠ 0. Then, (18) is simplified to Λ = *μ*_2_Λ_2_ + *μ*_3_Λ_3_ + *μ*_4_Λ_4_. Thus, $R_{10}=\Lambda_{2}+\kappa \Lambda_{3}+\sigma \Lambda_{4}$, *κ*, *σ* ≠ 0.

**Case 11:** *μ*_8_ = 0, *μ*_6_ = 0, *μ*_3_ = 0. By the adjoint action of Λ_4_, Λ_5_, Λ_6_ and Λ_8_, (18) reduces to Λ = *μ*_1_Λ_1_ + *e*^−*ϵ*^*μ*_7_Λ_7_. Therefore, $R_{11}=\Lambda_{1}\pm \Lambda_{7}$.

**Case 12:** *μ*_8_ = 0, *μ*_6_ = 0, *μ*_1_ = 0, *μ*_3_ ≠ 0. By the adjoint action of Λ_4_, Λ_5_ and Λ_8_ Eq (18) reduces to Λ = *μ*_2_Λ_2_ + *μ*_3_Λ_3_ + *e*^−*ϵ*^*μ*_7_Λ_7_. Therefore, $R_{12}=\Lambda_{2}+\kappa \Lambda_{3}\pm \Lambda_{7},\;\kappa \neq 0$.

**Case 13:** *μ*_8_ = 0, *μ*_6_ = 0, *μ*_1_ = 0, *μ*_2_ = 0, *μ*_3_ ≠ 0. By the adjoint action of Λ_4_, Λ_5_ and Λ_8_ (18), reduces to Λ = *μ*_3_Λ_3_ + *e*^−*ϵ*^*μ*_7_Λ_7_. Therefore, $R_{13}=\Lambda_{3}\pm \Lambda_{7}$.

**Case 14:** *μ*_8_ = 0, *μ*_6_ = 0, *μ*_1_ = 0, *μ*_2_ ≠ 0, *μ*_3_ = 0. By the adjoint action of Λ_4_, Λ_5_ and Λ_8_, (18) reduces to Λ = *μ*_2_Λ_2_ + *e*^−*ϵ*^*μ*_7_Λ_7_. Therefore, $R_{14}=\Lambda_{2}\pm \Lambda_{7}$.

**Case 15:** *μ*_8_ = 0, *μ*_1_ = 0, *μ*_3_ ≠ 0. By the adjoint action of Λ_1_, Λ_4_, Λ_5_ and Λ_8_ Eq (18) is reduced to Λ = *μ*_3_Λ_3_ + *e*^−*ϵ*^*μ*_6_Λ_6_ + *e*^−*ϵ*^*μ*_7_Λ_7_. Thus, $R_{15}=\Lambda_{3}\pm \Lambda_{6}\pm \Lambda_{7}$.

**Case 16:** *μ*_8_ = 0, *μ*_7_ = 0, *μ*_1_ = 0, *μ*_3_ ≠ 0. By the adjoint action of Λ_1_, Λ_7_ and Λ_8_, (18) reduces to Λ = *μ*_3_Λ_3_ + *e*^−*ϵ*^*μ*_6_Λ_6_. Therefore, $R_{16}=\Lambda_{3}\pm \Lambda_{6}$.

**Case 17:** *μ*_8_ = 0, *μ*_7_ = 0, *μ*_4_ = 0, *μ*_1_ = 0, *μ*_5_ ≠ 0. By the adjoint action of Λ_1_ and Λ_8_ Eq (18) reduces to Λ = *μ*_3_Λ_3_*μ*_5_Λ_5_ + *e*^−*ϵ*^*μ*_6_Λ_6_. Therefore, $R_{17}=\Lambda_{3}+\kappa \Lambda_{5}\pm \Lambda_{6},\;\kappa \neq 0$.

**Case 18:** *μ*_8_ = 0, *μ*_7_ = 0, *μ*_6_ = 0, *μ*_1_ = 0, *μ*_4_ = 0, *μ*_2_ = 0, *μ*_5_ ≠ 0. Then, (18) is reduced to Λ = *μ*_3_Λ_3_ + *μ*_5_Λ_5_. Therefore, $R_{18}=\Lambda_{3}+\kappa \Lambda_{5},\;\kappa \neq 0$.

**Case 19:** *μ*_8_ = 0, *μ*_7_ = 0, *μ*_6_ = 0, *μ*_1_ = 0, *μ*_5_ = 0, *μ*_2_ = 0, *μ*_4_ ≠ 0. Then, (18) is reduced to Λ = *μ*_3_Λ_3_ + *μ*_4_Λ_4_. Therefore, $R_{19}=\Lambda_{3}+\kappa \Lambda_{4},\;\kappa \neq 0$.

**Case 20:** *μ*_8_ = 0, *μ*_7_ = 0, *μ*_5_ = 0, *μ*_1_ = 0, *μ*_4_ ≠ 0. By the adjoint action of Λ_1_ and Λ_8_ Eq (18) reduces to Λ = *μ*_3_Λ_3_ + *μ*_4_Λ_4_ + *e*^−*ϵ*^*μ*_6_Λ_6_. Therefore, $R_{20}=\Lambda_{3}+\kappa \Lambda_{4}\pm \Lambda_{6},\;\kappa \neq 0$.

**Case 21:** *μ*_8_ = 0, *μ*_7_ = 0, *μ*_4_ = 0, *μ*_3_ = 0, *μ*_1_ = 0, *μ*_5_ ≠ 0. By the adjoint action of Λ_1_ and Λ_8_, (18) reduces to Λ = *μ*_5_Λ_5_ + *e*^−*ϵ*^*μ*_6_Λ_6_. Therefore, $R_{21}=\Lambda_{5}\pm \Lambda_{6}$.

**Case 22:** *μ*_8_ = 0, *μ*_7_ = 0, *μ*_4_ = 0, *μ*_3_ = 0, *μ*_1_ = 0, *μ*_5_ = 0. By the adjoint action of Λ_1_, (18) is reduced to Λ = *μ*_6_Λ_6_. Thus, $R_{22}=\Lambda_{6}$.

**Case 23:** *μ*_8_ = 0, *μ*_7_ = 0, *μ*_4_ = 0, *μ*_3_ = 0, *μ*_1_ = 0, *μ*_6_ = 0, *μ*_2_ = 0. Then, (18) is reduced to Λ = *μ*_5_Λ_5_. Therefore, $R_{23}=\Lambda_{5}$.

**Case 24:** *μ*_8_ = 0, *μ*_7_ ≠ 0, *μ*_3_ = 0, *μ*_1_ = 0. By the adjoint action of Λ_1_, Λ_4_ and Λ_5_, (18) reduces to Λ = *μ*_6_Λ_6_ + *μ*_7_Λ_7_. Therefore, $R_{24}=\Lambda_{6}+\kappa \Lambda_{7},\;\kappa \neq 0$.

**Case 25:** *μ*_8_ = 0, *μ*_7_ ≠ 0, *μ*_6_ = 0, *μ*_3_ = 0*μ*_2_ = 0, *μ*_1_ = 0. By the adjoint action of Λ_4_ and Λ_5_, Eq (18) is reduced to Λ = *μ*_7_Λ_7_. Thus, $R_{25}=\Lambda_{7}$.

**Case 26:** *μ*_8_ = 0, *μ*_7_ = 0, *μ*_5_ = 0, *μ*_4_ = 0, *μ*_3_ = 0. By the adjoint action of Λ_1_ and Λ_8_, Eq (18) is reduced to Λ = *μ*_1_Λ_1_ + *e*^−*ϵ*^*μ*_6_Λ_6_. Therefore, $R_{26}=\Lambda_{1}\pm \Lambda_{6}$.

**Case 27:** *μ*_8_ = 0, *μ*_7_ = 0, *μ*_6_ = 0, *μ*_5_ = 0, *μ*_4_ = 0, *μ*_3_ = 0. By the adjoint action of Λ_6_, (18) reduces to Λ = *μ*_1_Λ_1_. Thus, $R_{27}=\Lambda_{1}$.

**Case 28:** *μ*_8_ = 0, *μ*_7_ = 0, *μ*_5_ = 0, *μ*_3_ = 0, *μ*_1_ = 0, *μ*_6_ ≠ 0. By the adjoint action of Λ_1_ and Λ_8_, Eq (18) is reduced to Λ = *μ*_4_Λ_4_ + *e*^−*ϵ*^*μ*_6_Λ_6_. Therefore, $R_{28}=\Lambda_{4}\pm \Lambda_{6}$.

**Case 29:** *μ*_8_ = 0, *μ*_7_ = 0, *μ*_5_ = 0, *μ*_3_ = 0, *μ*_1_ = 0, *μ*_6_ = 0, *μ*_2_ = 0. Then, (18) is reduced to Λ = *μ*_4_Λ_4_. Thus, $R_{29}=\Lambda_{4}$.

**Case 30:** *μ*_8_ = 0, *μ*_7_ = 0, *μ*_6_ = 0, *μ*_4_ = 0, *μ*_3_ ≠ 0, *μ*_5_ ≠ 0. From the adjoint action of Λ_6_ and Λ_8_, Eq (18) reduces to Λ = *μ*_1_Λ_1_ + *μ*_3_Λ_3_ + *μ*_5_Λ_5_. Thus, $R_{30}=\Lambda_{1}+\kappa \Lambda_{3}+\sigma \Lambda_{5},\;\kappa ,\sigma \neq 0$,

**Case 31:** *μ*_8_ = 0, *μ*_7_ = 0, *μ*_6_ = 0, *μ*_4_ = 0, *μ*_3_ = 0, *μ*_5_ ≠ 0. By the adjoint action of Λ_6_, Eq (18) is reduced to Λ = *μ*_1_Λ_1_ + *μ*_5_Λ_5_. Therefore, $R_{31}=\Lambda_{1}+\kappa \Lambda_{5},\;\kappa \neq 0$.

**Case 32:** *μ*_8_ = 0, *μ*_7_ = 0, *μ*_6_ = 0, *μ*_4_ = 0, *μ*_1_ = 0, *μ*_3_ ≠ 0, *μ*_5_ ≠ 0. Then, (18) is reduced to Λ = *μ*_2_Λ_2_ + *μ*_3_Λ_3_ + *μ*_5_Λ_5_. Thus, $R_{32}=\Lambda_{2}+\kappa \Lambda_{3}+\sigma \Lambda_{5},\;\kappa ,\sigma \neq 0$,

**Case 33:** *μ*_8_ = 0, *μ*_7_ = 0, *μ*_6_ = 0, *μ*_4_ = 0, *μ*_1_ = 0, *μ*_3_ ≠ 0, *μ*_5_ = 0. Then, (18) is reduced to Λ = *μ*_2_Λ_2_ + *μ*_3_Λ_3_. Therefore, $R_{33}=\Lambda_{2}+\kappa \Lambda_{3},\;\kappa \neq 0$.

**Case 34:** *μ*_8_ = 0, *μ*_7_ = 0, *μ*_6_ = 0, *μ*_4_ = 0, *μ*_1_ = 0, *μ*_3_ = 0, *μ*_5_ ≠ 0. Then, (18) is reduced to Λ = *μ*_2_Λ_2_ + *μ*_5_Λ_5_. Therefore, $R_{34}=\Lambda_{2}+\kappa \Lambda_{5},\;\kappa \neq 0$.

**Case 35:** *μ*_8_ = 0, *μ*_7_ = 0, *μ*_6_ = 0, *μ*_4_ = 0, *μ*_1_ = 0, *μ*_3_ = 0, *μ*_5_ = 0. Then, (18) is reduced to Λ = *μ*_2_Λ_2_. Thus, $R_{35}=\Lambda_{2}$.

**Case 36:** *μ*_8_ = 0, *μ*_7_ = 0, *μ*_6_ = 0, *μ*_4_ = 0, *μ*_1_ = 0, *μ*_2_ = 0, *μ*_5_ = 0. Then, (18) is reduced to Λ = *μ*_3_Λ_3_. Thus, $R_{36}=\Lambda_{3}$.

**Case 37:** *μ*_8_ = 0, *μ*_7_ = 0, *μ*_6_ = 0, *μ*_5_ = 0, *μ*_3_ = 0, *μ*_4_ ≠ 0. By the adjoint action of Λ_6_, Eq (18) is reduced to Λ = *μ*_1_Λ_1_ + *μ*_4_Λ_4_. Therefore, $R_{37}=\Lambda_{1}+\kappa \Lambda_{4},\;\kappa \neq 0$.

**Case 38:** *μ*_8_ = 0, *μ*_7_ = 0, *μ*_6_ = 0, *μ*_5_ = 0, *μ*_3_ = 0, *μ*_1_ = 0, *μ*_4_ ≠ 0. Then, (18) is reduced to Λ = *μ*_2_Λ_2_ + *μ*_4_Λ_4_. Therefore, $R_{38}=\Lambda_{2}+\kappa \Lambda_{4},\;\kappa \neq 0$.

Hence, the one-dimensional optimal system for Eq (13) is given by,

$$\begin{array}{cc} \begin{array}{l} \begin{array}{c} R_{1}=\Lambda_{6}+\kappa \Lambda_{7}+\sigma \Lambda_{8},\;\kappa ,\sigma \neq 0 \end{array}\\ \begin{array}{c} R_{2}=\Lambda_{6}+\kappa \Lambda_{8},\;\kappa \neq 0 \end{array}\\ \begin{array}{c} R_{3}=\Lambda_{7}+\kappa \Lambda_{8},\;\kappa \neq 0 \end{array}\\ \begin{array}{c} R_{4}=\Lambda_{8} \end{array}\\ \begin{array}{c} R_{5}=\Lambda_{1}+\kappa \Lambda_{3}\pm \Lambda_{6}\pm \Lambda_{7},\;\kappa \neq 0 \end{array}\\ \begin{array}{c} R_{6}=\Lambda_{1}+\kappa \Lambda_{3}\pm \Lambda_{7},\;\kappa \neq 0 \end{array}\\ \begin{array}{c} R_{7}=\Lambda_{1}+\kappa \Lambda_{3}\pm \Lambda_{6},\;\kappa \neq 0 \end{array}\\ \begin{array}{c} R_{8}=\Lambda_{1}+\kappa \Lambda_{3},\;\kappa \neq 0 \end{array}\\ \begin{array}{c} R_{9}=\Lambda_{1}+\kappa \Lambda_{3}+\sigma \Lambda_{4},\;\kappa ,\sigma \neq 0 \end{array}\\ \begin{array}{c} R_{10}=\Lambda_{2}+\kappa \Lambda_{3}+\sigma \Lambda_{4},\;\kappa ,\sigma \neq 0 \end{array}\\ \begin{array}{c} R_{11}=\Lambda_{1}\pm \Lambda_{7} \end{array}\\ \begin{array}{c} R_{12}=\Lambda_{2}+\kappa \Lambda_{3}\pm \Lambda_{7},\;\kappa \neq 0 \end{array}\\ \begin{array}{c} R_{13}=\Lambda_{3}\pm \Lambda_{7} \end{array}\\ \begin{array}{c} R_{14}=\Lambda_{2}\pm \Lambda_{7} \end{array}\\ \begin{array}{c} R_{15}=\Lambda_{3}\pm \Lambda_{6}\pm \Lambda_{7} \end{array}\\ \begin{array}{c} R_{16}=\Lambda_{3}\pm \Lambda_{6} \end{array}\\ \begin{array}{c} R_{17}=\Lambda_{3}+\kappa \Lambda_{5}\pm \Lambda_{6},\;\kappa \neq 0 \end{array}\\ \begin{array}{c} R_{18}=\Lambda_{3}+\kappa \Lambda_{5},\;\kappa \neq 0 \end{array}\\ \begin{array}{c} R_{19}=\Lambda_{3}+\kappa \Lambda_{4},\;\kappa \neq 0 \end{array} \end{array} & \begin{array}{l} \begin{array}{c} R_{20}=\Lambda_{3}+\kappa \Lambda_{4}\pm \Lambda_{6},\;\kappa \neq 0 \end{array}\\ \begin{array}{c} R_{21}=\Lambda_{5}\pm \Lambda_{6} \end{array}\\ \begin{array}{c} R_{22}=\Lambda_{6} \end{array}\\ \begin{array}{c} R_{23}=\Lambda_{5} \end{array}\\ \begin{array}{c} R_{24}=\Lambda_{6}+\kappa \Lambda_{7},\;\kappa \neq 0 \end{array}\\ \begin{array}{c} R_{25}=\Lambda_{7} \end{array}\\ \begin{array}{c} R_{26}=\Lambda_{1}\pm \Lambda_{6} \end{array}\\ \begin{array}{c} R_{27}=\Lambda_{1} \end{array}\\ \begin{array}{c} R_{28}=\Lambda_{4}\pm \Lambda_{6} \end{array}\\ \begin{array}{c} R_{29}=\Lambda_{4} \end{array}\\ \begin{array}{c} R_{30}=\Lambda_{1}+\kappa \Lambda_{3}+\sigma \Lambda_{5},\;\kappa ,\sigma \neq 0 \end{array}\\ \begin{array}{c} R_{31}=\Lambda_{1}+\kappa \Lambda_{5},\;\kappa \neq 0 \end{array}\\ \begin{array}{c} R_{32}=\Lambda_{2}+\kappa \Lambda_{3}+\sigma \Lambda_{5},\;\kappa ,\sigma \neq 0 \end{array}\\ \begin{array}{c} R_{33}=\Lambda_{2}+\kappa \Lambda_{3},\;\kappa \neq 0 \end{array}\\ \begin{array}{c} R_{34}=\Lambda_{2}+\kappa \Lambda_{5},\;\kappa \neq 0 \end{array}\\ \begin{array}{c} R_{35}=\Lambda_{2} \end{array}\\ \begin{array}{c} R_{36}=\Lambda_{3} \end{array}\\ \begin{array}{c} R_{37}=\Lambda_{1}+\kappa \Lambda_{4},\;\kappa \neq 0 \end{array}\\ \begin{array}{c} R_{38}=\Lambda_{2}+\kappa \Lambda_{4},\;\kappa \neq 0 \end{array} \end{array} \end{array}$$

## 3 Group invariant solutions

Invariant solutions [19] are the solutions to differential equations that remain unchanged under the action of a given symmetry group. In the context of the Lie group method, invariant solutions are those that are preserved by the symmetry transformations generated by the infinitesimal generators of the group. In this section, we focus on investigating the invariant solutions of (13).

**Invariant Solutions by Using** $R_{27}=\Lambda_{1}.$

First, let us consider the infinitesimal generator $\Lambda_{1}=\frac{\partial }{\partial \tau }\cdot$

The characteristic equation associated with Λ_1_ is given by

$$\frac{dx_{1}}{0}=\frac{dx_{2}}{0}=\frac{dx_{3}}{0}=\frac{d\tau }{1}=\frac{dU}{0}\cdot$$

From the characteristic equation, we can deduce that *x*_1_ = *α*, *x*_2_ = *β*, *x*_3_ = λ, *U* = *X*(*α*, *β*, λ). Using these invariant variables, (13) is transformed into the equation given by

$$\begin{array}{c} \left(-CX_{\alpha }-A\right)X_{\alpha \alpha }+\left(-DX_{\alpha }-B\right)X_{\beta \beta }+\left(-DX_{\alpha }-B\right)X_{\lambda \lambda }-2D\left(X_{\beta }X_{\alpha \beta }+X_{\lambda }X_{\alpha \lambda }\right)=0, \end{array}$$ (19)

Infinitesimals of (19) are written as

$$\begin{array}{c} \begin{array}{cc} \delta_{\alpha } & =c_{2}\alpha +c_{5},\\ \delta_{\beta } & =-c_{1}\lambda +c_{2}\beta +c_{6},\\ \delta_{\lambda } & =c_{1}\beta +c_{2}\lambda +c_{3},\\ \Psi_{X} & =c_{2}X+c_{4}. \end{array} \end{array}$$ (20)

**Case 1:** By taking *c*_3_ = 1 and all other constants zero, the characteristic equation associated with (20) is

$$\frac{d\alpha }{0}=\frac{d\beta }{0}=\frac{d\lambda }{1}=\frac{dX}{0},$$

which implies that *α* = *r*, *β* = *s*, and *X*(*α*, *β*, λ) = *Y*(*r*, *s*). Using these invariant variables, (19) is transformed into the following equation

$$\begin{array}{c} \left(-DY_{r}-B\right)Y_{ss}+\left(-CY_{r}-A\right)Y_{rr}-2DY_{s}Y_{rs}=0. \end{array}$$ (21)

Infinitesimals of Eq (21) becomes

$$\begin{array}{c} \begin{array}{cc} \delta_{r} & =c_{1}r+c_{4},\\ \delta_{s} & =c_{1}s+c_{2},\\ \Psi_{Y} & =c_{1}Y+c_{3}. \end{array} \end{array}$$ (22)

**Case 1a:** By taking *c*_2_ = 1, *c*_4_ = 1 and all other constants zero, the characteristic equation associated with (22) is

$$\frac{dr}{1}=\frac{ds}{1}=\frac{dY}{0},$$

which implies that −*r* + *s* = *p* and *Y*(*r*, *s*) = *Z*(*p*). Using these invariant variables, (21) is transformed into the following ODE

$$\begin{array}{c} -Z^{\prime \prime }\left(A+B+\left(-C-3D\right)Z^{\prime }\right)=0. \end{array}$$ (23)

If *Z*″ = 0, then *Z*(*p*) = *c*_1_*p* + *c*_2_. This implies *Y*(*r*, *s*) = *c*_1_(*s* − *r*) + *c*_2_, this gives *X*(*α*, *β*, λ) = *c*_1_(*β* − *α*) + *c*_2_.

So, the solution of (13) in original variables is

$$U\left(x_{1},x_{2},x_{3},\tau \right)=c_{1}\left(x_{2}-x_{1}\right)+c_{2}.$$

Now, if *Z*″ ≠ 0, from (23), we have *A* + *B* + (−*C* − 3*D*)*Z*′ = 0 which gives $Z\left(p\right)=c_{1}+\frac{\left(A+B\right)}{\left(C+3D\right)}p,$ which provides $Y\left(r,s\right)=c_{1}+\frac{\left(A+B\right)}{\left(C+3D\right)}\left(s-r\right)$.

This implies,

$$\begin{array}{c} X\left(\alpha ,\beta ,\lambda \right)=c_{1}+\frac{\left(A+B\right)}{\left(C+3D\right)}\left(\beta -\alpha \right). \end{array}$$ (24)

Hence, the solution of (13) in original variables is

$$U\left(x_{1},x_{2},x_{3},\tau \right)=c_{1}+\frac{\left(A+B\right)}{\left(C+3D\right)}\left(x_{2}-x_{1}\right).$$

**Invariant Solutions by Using** $R_{29}=\Lambda_{4}.$

First, let us consider the infinitesimal generator $\Lambda_{4}=\frac{\partial }{\partial x_{2}}\cdot$

The characteristic equation associated with Λ_4_ is given by

$$\frac{dx_{1}}{0}=\frac{dx_{2}}{1}=\frac{dx_{3}}{0}=\frac{d\tau }{0}=\frac{dU}{0}\cdot$$

From the characteristic equation, we can deduce that *τ* = *α*, *x*_1_ = *β*, *x*_3_ = λ, *U* = *X*(*α*, *β*, λ). Using these invariant variables, (13) is transformed into the equation given by

$$\begin{array}{c} \left(-CX_{\beta }-A\right)X_{\beta \beta }+\left(-DX_{\beta }-B\right)X_{\lambda \lambda }-2DX_{\lambda }X_{\beta \lambda }+X_{\alpha \alpha }=0. \end{array}$$ (25)

Infinitesimals of (25) are written as

$$\begin{array}{c} \begin{array}{cc} \delta_{\alpha } & =c_{1}\alpha +c_{2},\\ \delta_{\beta } & =c_{1}\beta +c_{6},\\ \delta_{\lambda } & =c_{1}\lambda +c_{5},\\ \Psi_{X} & =c_{1}X+c_{3}\alpha +c_{4}. \end{array} \end{array}$$ (26)

**Case 2:** By taking *c*_4_ = 1, *c*_5_ = 1 and all other constants zero, the characteristic equation associated with (26) is

$$\frac{d\alpha }{0}=\frac{d\beta }{0}=\frac{d\lambda }{1}=\frac{dX}{1},$$

which implies that *α* = *r*, *β* = *s*, and *X*(*α*, *β*, λ) = λ + *Y*(*r*, *s*). Using these invariant variables, (25) is transformed into the following equation

$$\begin{array}{c} \left(-CY_{s}-A\right)Y_{ss}+Y_{rr}=0. \end{array}$$ (27)

Infinitesimals of Eq (27) becomes

$$\begin{array}{c} \begin{array}{cc} \delta_{r} & =c_{1}r+c_{2},\\ \delta_{s} & =c_{3}s+c_{4},\\ \Psi_{Y} & =\left(3c_{3}-2c_{1}\right)Y-\frac{2A}{C}\left(c_{1}-c_{3}\right)s+c_{5}r+c_{6}. \end{array} \end{array}$$ (28)

**Case 2a:** By taking *c*_2_ = 1, *c*_4_ = 1, *c*_6_ = 1 and all other constants zero, the characteristic equation associated with (28) is

$$\frac{dr}{1}=\frac{ds}{1}=\frac{dY}{1},$$

which implies that −*r* + *s* = *p* and *Y*(*r*, *s*) = *r* + *Z*(*p*). Using these invariant variables, (27) is transformed into the following ODE

$$\begin{array}{c} -Z^{\prime \prime }\left(CZ^{\prime }+A-1\right)=0. \end{array}$$ (29)

If *Z*″ = 0, then *Z*(*p*) = *c*_1_*p* + *c*_2_. This implies *Y*(*r*, *s*) = *c*_1_(*s* − *r*) + *c*_2_ + *r*, this gives *X*(*α*, *β*, λ) = *c*_1_(*β* − *α*) + *c*_2_ + *α* + λ.

So, the solution of (13) in original variables is

$$U\left(x_{1},x_{2},x_{3},\tau \right)=c_{1}\left(x_{1}-\tau \right)+c_{2}+\tau +x_{3}.$$

Now, if *Z*″ ≠ 0, then from (29), we have *CZ*′ + *A* − 1 = 0 which gives $Z\left(p\right)=c_{1}-\frac{\left(A-1\right)}{C}p,$ which provides $Y\left(r,s\right)=c_{1}+r-\frac{\left(A-1\right)}{C}\left(s-r\right)$.

This implies,

$$\begin{array}{c} X\left(\alpha ,\beta ,\lambda \right)=c_{1}+\alpha +\lambda -\frac{\left(A-1\right)}{C}\left(\beta -\alpha \right). \end{array}$$ (30)

Hence, the solution of (13) in original variables is

$$U\left(x_{1},x_{2},x_{3},\tau \right)=c_{1}+\tau +x_{3}-\frac{\left(A-1\right)}{C}\left(x_{1}-\tau \right).$$

**Invariant Solutions by Using** $R_{23}=\Lambda_{5}.$

First, let us consider the infinitesimal generator $\Lambda_{5}=\frac{\partial }{\partial x_{3}}\cdot$

The characteristic equation associated with Λ_5_ is given by

$$\frac{dx_{1}}{0}=\frac{dx_{2}}{0}=\frac{dx_{3}}{1}=\frac{d\tau }{0}=\frac{dU}{0}\cdot$$

From the characteristic equation, we can deduce that *τ* = *α*, *x*_1_ = *β*, *x*_2_ = λ, *U* = *X*(*α*, *β*, λ). Using these invariant variables, (13) is transformed into the equation given by

$$\begin{array}{c} \left(-CX_{\beta }-A\right)X_{\beta \beta }+\left(-DX_{\beta }-B\right)X_{\lambda \lambda }-2DX_{\lambda }X_{\beta \lambda }+X_{\alpha \alpha }=0. \end{array}$$ (31)

Infinitesimals of (31) are written as

$$\begin{array}{c} \begin{array}{cc} \delta_{\alpha } & =c_{1}\alpha +c_{2},\\ \delta_{\beta } & =c_{1}\beta +c_{6},\\ \delta_{\lambda } & =c_{1}\lambda +c_{5},\\ \Psi_{X} & =c_{1}X+c_{3}\alpha +c_{4}. \end{array} \end{array}$$ (32)

**Case 3:** By taking *c*_2_ = 1, *c*_4_ = 1 and all other constants zero, the characteristic equation associated with (32) is

$$\frac{d\alpha }{1}=\frac{d\beta }{0}=\frac{d\lambda }{0}=\frac{dX}{1},$$

which implies that *β* = *r*, λ = *s*, and *X*(*α*, *β*, λ) = *α* + *Y*(*r*, *s*). Using these invariant variables, (31) is transformed into the following equation

$$\begin{array}{c} \left(-DY_{r}-B\right)Y_{ss}+\left(-CY_{r}-A\right)Y_{rr}-2DY_{s}Y_{rs}=0. \end{array}$$ (33)

Infinitesimals of Eq (33) becomes

$$\begin{array}{c} \begin{array}{cc} \delta_{r} & =c_{1}r+c_{4},\\ \delta_{s} & =c_{1}s+c_{2},\\ \Psi_{Y} & =c_{1}Y+c_{3}. \end{array} \end{array}$$ (34)

**Case 3a:** By taking *c*_4_ = 1 and all other constants zero, the characteristic equation associated with (34) is

$$\frac{dr}{1}=\frac{ds}{0}=\frac{dY}{0},$$

which implies that *s* = *p* and *Y*(*r*, *s*) = *Z*(*p*). Using these invariant variables, (33) is transformed into the following ODE

$$\begin{array}{c} -BZ^{\prime \prime }=0. \end{array}$$ (35)

This implies *Z*(*p*) = *c*_1_*p* + *c*_2_, this gives *Y*(*r*, *s*) = *c*_1_*s* + *c*_2_,

This implies,

$$\begin{array}{c} X\left(\alpha ,\beta ,\lambda \right)=c_{1}\lambda +c_{2}+\alpha , \end{array}$$ (36)

So, the solution of (13) in original variables is

$$U\left(x_{1},x_{2},x_{3},\tau \right)=c_{1}x_{2}+c_{2}+\tau .$$

**Invariant Solutions by Using** $R_{8}=\Lambda_{1}+\kappa \Lambda_{3}.$

First, let us consider the infinitesimal generator $\Lambda_{1}+\kappa \Lambda_{3}=\frac{\partial }{\partial \tau }+\kappa \frac{\partial }{\partial x_{1}}\cdot$

The characteristic equation associated with Λ_1_ + *κ*Λ_3_ is given by

$$\frac{dx_{1}}{\kappa }=\frac{dx_{2}}{0}=\frac{dx_{3}}{0}=\frac{d\tau }{1}=\frac{dU}{0}\cdot$$

From the characteristic equation, we deduce that $x_{2}=\alpha ,\;x_{3}=\beta ,\;\tau -\frac{x_{1}}{\kappa }=\lambda ,\;U=X\left(\alpha ,\beta ,\lambda \right)$. Using these invariant variables, (13) is transformed into the equation given by

$$\begin{array}{c} \begin{array}{c} -B\kappa^{3}X_{\alpha \alpha }-B\kappa^{3}X_{\beta \beta }+\kappa^{2}DX_{\lambda }X_{\alpha \alpha }+\kappa^{2}DX_{\lambda }X_{\beta \beta }+2\kappa^{2}DX_{\beta }X_{\beta \lambda }+2\kappa^{2}DX_{\alpha }X_{\alpha \lambda }\\ +\kappa^{3}X_{\lambda \lambda }-\kappa AX_{\lambda \lambda }+CX_{\lambda }X_{\lambda \lambda }=0, \end{array} \end{array}$$ (37)

Infinitesimals of (37) are written as

$$\begin{array}{c} \begin{array}{cc} \delta_{\alpha } & =c_{1}\beta +c_{2}\alpha +c_{3},\\ \delta_{\beta } & =-c_{1}\alpha +c_{2}\beta +c_{6},\\ \delta_{\lambda } & =c_{2}\lambda +c_{5},\\ \Psi_{X} & =c_{2}X+c_{4}. \end{array} \end{array}$$ (38)

**Case 4:** By taking *c*_3_ = 1, *c*_6_ = 1 and all other constants zero, the characteristic equation associated with (38) is

$$\frac{d\alpha }{1}=\frac{d\beta }{1}=\frac{d\lambda }{0}=\frac{dX}{0},$$

which implies that λ = *r*, *β* − *α* = *s* and *X*(*α*, *β*, λ) = λ + *Y*(*r*, *s*). Using these invariant variables, (37) is transformed into the following equation

$$\begin{array}{c} \left(\kappa^{3}-\kappa A+CY_{r}\right)Y_{rr}+\left(-2B\kappa^{3}+2D\kappa^{2}Y_{r}\right)Y_{ss}+4D\kappa^{2}Y_{s}Y_{rs}=0, \end{array}$$ (39)

Infinitesimals of Eq (39) becomes

$$\begin{array}{c} \begin{array}{cc} \delta_{r} & =c_{1}r+c_{4},\\ \delta_{s} & =c_{1}s+c_{2},\\ \Psi_{Y} & =c_{1}Y+c_{3}. \end{array} \end{array}$$ (40)

**Case 4a:** By taking *c*_2_ = 1, *c*_3_ = 1, *c*_4_ = 1 and all other constants zero, the characteristic equation associated with (40) is

$$\frac{dr}{1}=\frac{ds}{1}=\frac{dY}{1},$$

which implies that −*r* + *s* = *p*, *Y*(*r*, *s*) = *r* + *Z*(*p*). Using these invariant variables, (39) is transformed into the following ODE

$$\begin{array}{c} -Z^{\prime \prime }\left(\left(6D\kappa^{2}+C\right)Z^{\prime }+\left(2B-1\right)\kappa^{3}-2D\kappa^{2}+\kappa A-C\right)=0, \end{array}$$ (41)

If *Z*″ = 0, then *Z*(*p*) = *c*_1_*p* + *c*_2_. This implies *Y*(*r*, *s*) = *c*_1_(*s* − *r*) + *c*_2_ + *r*, this gives *X*(*α*, *β*, λ) = *c*_1_(*β* − *α* − λ) + *c*_2_ + 2λ.

So, the solution of (13) in original variables is

$$U\left(x_{1},x_{2},x_{3},\tau \right)=c_{1}(x_{3}-x_{2}-(\tau -\frac{x_{1}}{\kappa }))+c_{2}+2(\tau -\frac{x_{1}}{\kappa }).$$

Now, if *Z*″ ≠ 0, then from (41), we have (6*Dκ*^2^ + *C*)*Z*′ + (2*B* − 1)*κ*^3^ − 2*Dκ*^2^ + *κA* − *C* = 0, which yields $Z\left(p\right)=\frac{(2D\kappa^{2}-\kappa A+C-\left(2B-1\right)\kappa^{3})p}{6D\kappa^{2}+C}+c_{1},$ which provides $Y\left(r,s\right)=\frac{(2D\kappa^{2}-\kappa A+C-\left(2B-1\right)\kappa^{3})\left(s-r\right)}{6D\kappa^{2}+C}+c_{1}+r$.

This implies,

$$\begin{array}{c} X\left(\alpha ,\beta ,\lambda \right)=\frac{(2D\kappa^{2}-\kappa A+C-\left(2B-1\right)\kappa^{3})\left(\beta -\alpha -\lambda \right)}{6D\kappa^{2}+C}+c_{1}+2\lambda . \end{array}$$ (42)

Hence, the solution of (13) in original variables is

$$U\left(x_{1},x_{2},x_{3},\tau \right)=\frac{\left(2D\kappa^{2}-\kappa A+C-\left(2B-1\right)\kappa^{3}\right)(x_{3}-x_{2}-(\tau -\frac{x_{1}}{\kappa }))}{6D\kappa^{2}+C}+c_{1}+2(\tau -\frac{x_{1}}{\kappa }).$$

**Invariant Solutions by Using** $R_{16}=\Lambda_{3}+\Lambda_{6}.$

First, let us consider the infinitesimal generator $\Lambda_{3}+\Lambda_{6}=\frac{\partial }{\partial x_{1}}+\tau \frac{\partial }{\partial U}\cdot$

The characteristic equation associated with Λ_3_ + Λ_6_ is given by

$$\frac{dx_{1}}{1}=\frac{dx_{2}}{0}=\frac{dx_{3}}{0}=\frac{d\tau }{0}=\frac{dU}{\tau }\cdot$$

From the characteristic equation, we can deduce that *τ* = *α*, *x*_2_ = *β*, *x*_3_ = λ, *U* = *x*_1_*τ* + *X*(*α*, *β*, λ). Using these invariant variables, (13) is transformed into the equation given by

$$\begin{array}{c} \left(-\alpha D-B\right)X_{\beta \beta }+\left(-\alpha D-B\right)X_{\lambda \lambda }+X_{\alpha \alpha }=0, \end{array}$$ (43)

Infinitesimals of (43) are written as

$$\begin{array}{c} \begin{array}{cc} \delta_{\alpha } & =\frac{2\left(\alpha D+B\right)(\left(c_{2}\beta +c_{4}\right)D+\frac{9}{2}c_{1}\lambda )}{3D^{2}},\\ \delta_{\beta } & =\frac{2}{9D^{2}}c_{2}{\left(\alpha D+B\right)}^{3}+\frac{c_{2}}{2}\beta^{2}+c_{4}\beta +\frac{9}{2D}c_{1}\beta \lambda -\frac{c_{2}}{2}\lambda^{2}-c_{3}\lambda +c_{13},\\ \delta_{\lambda } & =-\frac{9}{4D}c_{1}\beta^{2}+\left(c_{2}\lambda +c_{3}\right)\beta +\frac{9}{4D}c_{1}\lambda^{2}+c_{4}\lambda +c_{5}+c_{1}{\left(\alpha +\frac{B}{D}\right)}^{3},\\ \Psi_{X} & =\frac{1}{3De^{\sqrt{a_{3}}\lambda }e^{\sqrt{a_{2}}\beta }}(3Dc_{7}(c_{11}{\left(e^{\sqrt{a_{3}}\lambda }\right)}^{2}+c_{12})({\left(e^{\sqrt{a_{2}}\beta }\right)}^{2}c_{9}+c_{10})AiryAi(-\\ & \frac{\left(\alpha D+B\right){\left(-D\left(a_{2}+a_{3}\right)\right)}^{\frac{1}{3}}}{D})+3Dc_{8}(c_{11}{\left(e^{\sqrt{a_{3}}\lambda }\right)}^{2}+c_{12})({\left(e^{\sqrt{a_{2}}\beta }\right)}^{2}c_{9}+c_{10})AiryBi\\ & (-\frac{\left(\alpha D+B\right){\left(-D\left(a_{2}+a_{3}\right)\right)}^{\frac{1}{3}}}{D})-2e^{\sqrt{a_{2}}\beta }e^{\sqrt{a_{3}}\lambda }X(\left(c_{2}\beta -\frac{3}{2}c_{6}\right)D+\frac{9}{2}c_{1}\lambda )). \end{array} \end{array}$$ (44)

**Case 5:** By taking *c*_13_ = 1 and all other constants zero, the characteristic equation associated with (44) is

$$\frac{d\alpha }{0}=\frac{d\beta }{0}=\frac{d\lambda }{1}=\frac{dX}{0},$$

which implies that *α* = *r*, *β* = *s*, *X*(*α*, *β*, λ) = *Y*(*r*, *s*). Using these invariant variables, (43) is transformed into the following equation

$$\begin{array}{c} \left(-rD-B\right)Y_{ss}+Y_{rr}=0. \end{array}$$ (45)

Infinitesimals of Eq (45) becomes

$$\begin{array}{c} \begin{array}{cc} \delta_{r} & =\frac{\left(rD+B\right)\left(c_{7}D-4c_{1}s\right)}{D},\\ \delta_{s} & =-\frac{4}{3D^{2}}c_{1}{\left(rD+B\right)}^{3}-3c_{1}s^{2}+\frac{3}{2}Dc_{7}s+c_{8},\\ \Psi_{Y} & =\frac{1}{e^{\sqrt{a_{2}}s}}(c_{3}(c_{5}{\left(e^{\sqrt{a_{2}}s}\right)}^{2}+c_{6})AiryAi(-\frac{{\left(-a_{2}D\right)}^{\frac{1}{3}}\left(rD+B\right)}{D})+c_{4}(c_{5}{\left(e^{\sqrt{a_{2}}s}\right)}^{2}+c_{6})AiryBi\\ & (-\frac{{\left(-a_{2}D\right)}^{\frac{1}{3}}\left(rD+B\right)}{D})+e^{\sqrt{a_{2}}s}Y\left(c_{1}s+c_{2}\right)). \end{array} \end{array}$$ (46)

**Case 5a:** By taking *c*_7_ = 1 and all other constants zero, the characteristic equation associated with (46) is

$$\frac{dr}{\left(rD+B\right)}=\frac{ds}{\frac{3}{2}sD}=\frac{dY}{0},$$

which implies that $\frac{s}{{\left(rD+B\right)}^{\frac{3}{2}}}=p$ and *Y*(*r*, *s*) = *Z*(*p*). Using these invariant variables, (45) is transformed into the following ODE

$$\begin{array}{c} 9D^{2}p^{2}Z^{\prime \prime }+15D^{2}pZ^{\prime }-4Z^{\prime \prime }=0. \end{array}$$ (47)

This implies,

$$\begin{array}{c} Z\left(p\right)=c_{1}+hypergeom([\frac{1}{2},\frac{5}{6}],[\frac{3}{2}],\frac{9}{4}D^{2}p^{2})c_{2}p, \end{array}$$ (48)

which gives,

$$\begin{array}{c} Y\left(r,s\right)=c_{1}+hypergeom([\frac{1}{2},\frac{5}{6}],[\frac{3}{2}],\frac{9}{4}D^{2}\frac{s^{2}}{{\left(rD+B\right)}^{3}})c_{2}\frac{s}{{\left(rD+B\right)}^{\frac{3}{2}}}, \end{array}$$ (49)

this gives,

$$\begin{array}{c} X\left(\alpha ,\beta ,\lambda \right)=c_{1}+hypergeom([\frac{1}{2},\frac{5}{6}],[\frac{3}{2}],\frac{9}{4}D^{2}\frac{\beta^{2}}{{\left(\alpha D+B\right)}^{3}})c_{2}\frac{\beta }{{\left(\alpha D+B\right)}^{\frac{3}{2}}}, \end{array}$$ (50)

So, the solution of (13) in original variables is

$$\begin{array}{c} U\left(x_{1},x_{2},x_{3},\tau \right)=c_{1}+x_{1}\tau +hypergeom([\frac{1}{2},\frac{5}{6}],[\frac{3}{2}],\frac{9}{4}D^{2}\frac{x_{2}^{2}}{{\left(\tau D+B\right)}^{3}})c_{2}\frac{x_{2}}{{\left(\tau D+B\right)}^{\frac{3}{2}}}. \end{array}$$ (51)

**Invariant Solutions by Using** $R_{7}=\Lambda_{1}+\kappa \Lambda_{3}+\Lambda_{6}.$

First, let us consider the infinitesimal generator $\Lambda_{1}+\kappa \Lambda_{3}+\Lambda_{6}=\frac{\partial }{\partial \tau }+\kappa \frac{\partial }{\partial x_{1}}+\tau \frac{\partial }{\partial U}\cdot$

The characteristic equation associated with Λ_1_ + *κ*Λ_3_ + Λ_6_ is given by

$$\frac{dx_{1}}{\kappa }=\frac{dx_{2}}{0}=\frac{dx_{3}}{0}=\frac{d\tau }{1}=\frac{dU}{\tau }\cdot$$

From the characteristic equation, we deduce that $x_{2}=\alpha ,\;x_{3}=\beta ,\;\tau -\frac{x_{1}}{\kappa }=\lambda ,\;U=\frac{2\kappa x_{1}\tau -x_{1}^{2}}{2\kappa^{2}}+X\left(\alpha ,\beta ,\lambda \right)$. Using these invariant variables, (13) is transformed into the equation given by

$$\begin{array}{c} \begin{array}{c} \left(\kappa^{3}-A\kappa +CX_{\lambda }-C\lambda \right)X_{\lambda \lambda }+\kappa^{2}\left(DX_{\lambda }-B\kappa -D\lambda \right)X_{\beta \beta }+\kappa^{2}\left(DX_{\lambda }-B\kappa -D\lambda \right)X_{\alpha \alpha }\\ +2D\kappa^{2}X_{\alpha }X_{\alpha \lambda }+2D\kappa^{2}X_{\beta }X_{\beta \lambda }+A\kappa -CX_{\lambda }+C\lambda =0, \end{array} \end{array}$$ (52)

Infinitesimals of (52) are written as

$$\begin{array}{c} \begin{array}{cc} \delta_{\alpha } & =c_{1}\beta +c_{2},\\ \delta_{\beta } & =-c_{1}\alpha +c_{5},\\ \delta_{\lambda } & =c_{3},\\ \Psi_{X} & =c_{3}\lambda +c_{4}. \end{array} \end{array}$$ (53)

**Case 6:** By taking *c*_3_ = 1 and all other constants zero, the characteristic equation associated with (53) is

$$\frac{d\alpha }{0}=\frac{d\beta }{0}=\frac{d\lambda }{1}=\frac{dX}{\lambda },$$

which implies that *α* = *r*, *β* = *s*, and $X\left(\alpha ,\beta ,\lambda \right)=\frac{\lambda^{2}}{2}+Y\left(r,s\right)$. Using these invariant variables, (52) is transformed into the following equation

$$\begin{array}{c} -\kappa^{3}\left(BY_{ss}+BY_{rr}-1\right)=0. \end{array}$$ (54)

Infinitesimals of Eq (54) becomes

$$\begin{array}{c} \begin{array}{cc} \delta_{r} & =B\left(F_{4}\left(s-Ir\right)+F_{6}\left(s+Ir\right)\right)-c_{2}s+\frac{c_{1}}{2}r+c_{4},\\ \delta_{s} & =-BI\left(-F_{6}\left(s+Ir\right)+F_{4}\left(s-Ir\right)\right)+\frac{c_{1}}{2}s+c_{2}r+c_{3},\\ \Psi_{Y} & =c_{1}Y+F_{3}\left(s-Ir\right)+rF_{4}\left(s-Ir\right)+F_{5}\left(s+Ir\right)+rF_{6}\left(s+Ir\right). \end{array} \end{array}$$ (55)

**Case 6a:** By taking *c*_3_ = 1, *c*_4_ = 1 and all other constants zero, the characteristic equation associated with (55) is

$$\frac{dr}{1}=\frac{ds}{1}=\frac{dY}{0},$$

which implies that −*r* + *s* = *p*, *Y*(*r*, *s*) = *Z*(*p*). Using these invariant variables, (54) is transformed into the following ODE

$$\begin{array}{c} -2B\kappa^{3}Z^{\prime \prime }+\kappa^{3}=0. \end{array}$$ (56)

This implies,

$$\begin{array}{c} Z\left(p\right)=\frac{1}{4B}p^{2}+c_{1}p+c_{2}, \end{array}$$ (57)

which yields,

$$\begin{array}{c} Y\left(r,s\right)=\frac{1}{4B}{\left(-r+s\right)}^{2}+c_{1}\left(-r+s\right)+c_{2}, \end{array}$$ (58)

this gives,

$$\begin{array}{c} X\left(\alpha ,\beta ,\lambda \right)=\frac{\lambda^{2}}{2}+\frac{1}{4B}{\left(-\alpha +\beta \right)}^{2}+c_{1}\left(-\alpha +\beta \right)+c_{2}. \end{array}$$ (59)

So, the solution of (13) in original variables is

$$\begin{array}{c} U\left(x_{1},x_{2},x_{3},\tau \right)=\frac{2\kappa x_{1}\tau -x_{1}^{2}}{2\kappa^{2}}+\frac{{\left(\tau -\frac{x_{1}}{\kappa }\right)}^{2}}{2}+\frac{1}{4B}{\left(-x_{2}+x_{3}\right)}^{2}+c_{1}\left(-x_{2}+x_{3}\right)+c_{2}. \end{array}$$ (60)

## 4 Noether’s approach

Let us contemplate a differential system of *m*-th order

$$\begin{array}{c} G(x,U,U^{\tau },U^{\left(1\right)},U^{\left(2\right)}\cdots ,U^{\left(m\right)})=0, \end{array}$$ (61)

where *x*^*i*^ with *i* = 1, 2, ⋯, *n* represents independent variables, and *U*^*α*^ with *α* = 1, 2, ⋯, *m* symbolizes the dependent variables. Additionally, the notation *U*^*m*^ signifies the *m* th-order partial derivative.

The Lie-Bäcklund operator is defined as follows

$$\begin{array}{c} \Lambda_{1}=\delta^{i}\frac{\partial }{\partial x^{i}}+\Psi^{\varpi }\frac{\partial }{\partial U^{\varpi }}+\sum_{s⩾1}\Psi_{i_{1}\cdots i_{s}}^{\varpi }\frac{\partial }{\partial U_{i_{1}\cdots i_{s}}^{\varpi }}, \end{array}$$ (62)

where $\Psi_{i_{1}\cdots i_{s}}^{\varpi }$ is defined by

$$\begin{array}{c} \Psi_{i}^{\varpi }=D_{i}(\Psi^{\varpi })-U_{j}^{\varpi }D_{i}(\delta^{j}),\\ \Psi_{i_{1}\cdots i_{s}}^{\varpi }=D_{i_{s}}(\Psi_{i_{1}\cdots i_{s-1}}^{\varpi })-U_{ji_{1}\cdots i_{s-1}}^{\varpi }D_{i_{s}}(\delta^{j}),\;s>1, \end{array}$$

In this context, **D**_*i*_ denotes the total derivative operator.

The Lie-Bäcklund operator (62), when expressed in characteristic form, takes on the following representation

$$\begin{array}{c} \Lambda =\delta^{i}\frac{\partial }{\partial x^{i}}+W^{\varpi }\frac{\partial }{\partial U^{\varpi }}+D_{i}(W^{\varpi })\frac{\partial }{\partial U_{i}^{\varpi }}+D_{i}D_{j}(W^{\varpi })\frac{\partial }{\partial U_{ij}^{\varpi }}+\cdots , \end{array}$$ (63)

where,

$$W^{\varpi }=\Psi^{\delta }-\delta^{j}U_{j}^{\varpi },\;\varpi =1,2,\ldots ,m,$$

constitute Lie characteristic functions.

The Noether operators linked with a Lie-Bäcklund operator Λ are given by

$$\begin{array}{c} N^{i}=\Psi^{i}+W^{\varpi }\frac{\delta }{\delta U_{i}^{\varpi }}+\sum_{s⩾1}D_{i_{1}}\cdots D_{i_{s}}(W^{\varpi })\frac{\delta }{\delta U_{ij_{1}\ldots j_{s}}^{\varpi }},\;i=1,2,\ldots ,n, \end{array}$$ (64)

where $\frac{\delta }{\delta U_{i}^{\varpi }}$ denotes the Euler-Lagrange operator, which is defined as

$$\begin{array}{c} \frac{\delta }{\delta U_{i}^{\varpi }}=\frac{\partial }{\partial U_{i}^{\varpi }}+\sum_{s⩾1}{\left(-1\right)}^{s}D_{j_{1}}\cdots D_{j_{s}}\frac{\partial }{\partial U_{ij_{1}\ldots j_{s}}^{\varpi }},\;i=1,2,\ldots ,n,\;\varpi =1,2,\cdots ,m. \end{array}$$ (65)

**Lagrangian** A Lagrangian for the Eq (61) is described by a function $L=L(x,U,U^{\left(1\right)},\cdots ,U^{\left(m-1\right)})$, which adheres to the Euler-Lagrange equation

$$\begin{array}{c} \frac{\delta L}{\delta U^{\alpha }}=0. \end{array}$$ (66)

**Noether Symmetry Generator** A Lie-Bäcklund operator Λ is considered a Noether symmetry generator of the Eq (61) with respect to a Lagrangian L if there exist gauge functions $G^{i}=\left(G^{1},\cdots ,G^{m}\right)$ that fulfill the condition

$$\begin{array}{c} \Lambda \left(L\right)+LD_{i}\left(\Psi^{i}\right)=D_{i}\left(G^{i}\right). \end{array}$$ (67)

**Noether Conserved Vectors** Each Noether symmetry generator Λ associated with a given Lagrangian L that relates to the Euler-Lagrange differential equations corresponds to a vector *T* = (*T*^1^, *T*^2^, ⋯, *T*^*m*^). The definition of each component *T*^*i*^ is as follows

$$\begin{array}{c} T^{i}=G^{i}-N^{i}L=G^{i}-\delta^{i}L-W^{\varpi }\frac{\delta L}{\delta U_{i}^{\varpi }}-\sum_{s⩾1}D_{i_{1}\cdots i_{s}}(W^{\varpi })\frac{\delta L}{\delta U_{i_{1}\cdots i_{s}}^{\varpi }}, \end{array}$$ (68)

Vector *T*^*i*^ serves as a conserved quantity for Eq (66). Eq (67) is employed to identify the Noether symmetries, whereas (68) provides the associated Noether conserved vectors.

### 4.1 Noether’s approach to Eq (13)

In this subsection, we utilize Noether’s approach [20] to determine the conservation laws of (13). We begin by determining the Noether symmetries, and then establish the corresponding conservation laws using the Noether theorem.

The Lagrangian for Eq (13) given by

$$\begin{array}{c} L=\frac{AU_{x_{1}}^{2}}{2}+\frac{B}{2}\left(U_{x_{2}}^{2}+U_{x_{3}}^{2}\right)+\frac{CU_{x_{1}}^{3}}{6}-\frac{U_{\tau }^{2}}{2}+\frac{D}{2}\left(U_{x_{1}}U_{x_{2}}^{2}+U_{x_{1}}U_{x_{3}}^{2}\right). \end{array}$$ (69)

Applying the Euler operator (65) to the Eq (69), we get

$$\begin{array}{c} \frac{\delta L}{\delta u}=U_{\tau \tau }-AU_{x_{1}x_{1}}-B\left(U_{x_{2}x_{2}}+U_{x_{3}x_{3}}\right)-CU_{x_{1}}U_{x_{1}x_{1}}-D\left(U_{x_{1}}U_{x_{2}x_{2}}+U_{x_{1}}U_{x_{3}x_{3}}\right)\\ -2D(U_{x_{2}}U_{x_{1}x_{2}}+U_{x_{3}}U_{x_{1}x_{3}})=0. \end{array}$$ (70)

Hence, we can deduce Noether symmetries by employing the calculated Lagrangian. These symmetries, identified through Noether’s theorem, enable us to establish the conservation laws associated with Eq (13). These conservation laws offer valuable insights into the underlying physics and behavior of the system.

#### 4.1.1 Computation of the noether symmetries

We consider the following vector field

$$\Lambda =\delta_{1}\frac{\partial }{\partial x_{1}}+\delta_{2}\frac{\partial }{\partial x_{2}}+\delta_{3}\frac{\partial }{\partial x_{3}}+\delta_{4}\frac{\partial }{\partial \tau }+\Psi \frac{\partial }{\partial U}\cdot$$

The vector field Λ is called a Noether symmetry of Eq (13) corresponding to Lagrangian (69) if it fulfills the condition

$$\begin{array}{c} \Lambda^{\left[1\right]}L+L\left(D_{\tau }\delta_{4}+D_{x_{1}}\delta_{1}+D_{x_{2}}\delta_{2}+D_{x_{3}}\delta_{3}\right)=D_{\tau }G_{1}+D_{x_{1}}G_{2}+D_{x_{2}}G_{3}+D_{x_{3}}G_{4}, \end{array}$$ (71)

where,

$$\Lambda^{\left[1\right]}=\Lambda +\Psi^{x_{1}}\frac{\partial }{\partial U_{x_{1}}}+\Psi^{x_{2}}\frac{\partial }{\partial U_{x_{2}}}+\Psi^{x_{3}}\frac{\partial }{\partial U_{x_{3}}}+\Psi^{\tau }\frac{\partial }{\partial U_{\tau }},$$

and $G_{i}\left(x_{1},x_{2},x_{3},\tau ,U\right),i=1,2,3,4$ are gauge terms. We compare the coefficients of derivatives of *U* in the above equation to obtain the determining system. After performing some steps we get

$$\begin{array}{c} \begin{array}{cc} & A\delta_{2_{x_{2}}}-A\delta_{1_{x_{1}}}+A\delta_{3_{x_{3}}}+A\delta_{4_{\tau }}+2A\Psi_{U}+C\Psi_{x_{1}}=0,\\ & B\delta_{1_{x_{1}}}-B\delta_{2_{x_{2}}}+B\delta_{3_{x_{3}}}+B\delta_{4_{\tau }}+2B\Psi_{U}+D\Psi_{x_{1}}=0,\\ & B\delta_{1_{x_{1}}}+B\delta_{2_{x_{2}}}-B\delta_{3_{x_{3}}}+B\delta_{4_{\tau }}+2B\Psi_{U}+D\Psi_{x_{1}}=0,\\ & 2\Psi_{U}+\delta_{1_{x_{1}}}+\delta_{2_{x_{2}}}+\delta_{3_{x_{3}}}-\delta_{4_{\tau }}=0,\\ & 3\Psi_{U}-2\delta_{1_{x_{1}}}+\delta_{2_{x_{2}}}+\delta_{3_{x_{3}}}+\delta_{4_{\tau }}=0,\\ & 3\Psi_{U}-\delta_{2_{x_{2}}}+\delta_{3_{x_{3}}}+\delta_{4_{\tau }}=0,\\ & 3\Psi_{U}+\delta_{2_{x_{2}}}-\delta_{3_{x_{3}}}+\delta_{4_{\tau }}=0,\\ & \delta_{1_{x_{2}}}=\delta_{1_{x_{3}}}=\delta_{1_{\tau }}=\delta_{1_{U}}=0,\\ & \delta_{2_{x_{1}}}=\delta_{2_{\tau }}=\delta_{2_{U}}=0,\\ & \delta_{2_{x_{3}}}+\delta_{3_{x_{2}}}=0,\\ & \delta_{3_{x_{1}}}=\delta_{3_{\tau }}=\delta_{3_{U}}=0,\\ & \delta_{4_{x_{1}}}=\delta_{4_{x_{2}}}=\delta_{4_{x_{3}}}=\delta_{4_{U}}=0,\\ & \Psi_{x_{2}}=\Psi_{x_{3}}=G_{U}^{3}=G_{U}^{4}=0,\\ & A\Psi_{x_{1}}-G_{U}^{2}=0,\\ & -\Psi_{\tau }-G_{U}^{1}=0,\\ & G_{\tau }^{1}+G_{x_{1}}^{2}+G_{x_{2}}^{3}+G_{x_{3}}^{4}=0. \end{array} \end{array}$$ (72)

The following result follows

$$\begin{array}{c} \begin{array}{cc} & \delta_{1}=C_{1},\;\delta_{2}=C_{3}x_{3}+C_{4},\;\delta_{3}=-C_{3}x_{2}+C_{5},\;\delta_{4}=C_{2},\;\Psi =C_{6}\tau +C_{7},\\ & G^{1}=-C_{6}U+X_{x_{1}},\;G^{2}=-X_{\tau }+\int \left(-G_{x_{2}}-h_{x_{3}}\right)dx_{1}+p\left(x_{2},x_{3},\tau \right),\\ & G^{3}=g\left(x_{1},x_{2},x_{3},\tau \right),\;G^{4}=h\left(x_{1},x_{2},x_{3},\tau \right). \end{array} \end{array}$$ (73)

By taking *f* = *g* = *h* = *p* = 0.

then,

$$G^{1}=-C_{6}U,\;G^{2}=G^{3}=G^{4}=0.$$

Finally, we get the following Noether symmetry generators

$$\Lambda_{1}=\frac{\partial }{\partial x_{1}},\;\Lambda_{2}=\frac{\partial }{\partial x_{2}},\;\Lambda_{3}=\frac{\partial }{\partial x_{3}},\;\Lambda_{4}=\frac{\partial }{\partial \tau },\;\Lambda_{5}=\frac{\partial }{\partial U},$$

$$\Lambda_{6}=x_{3}\frac{\partial }{\partial x_{2}}-x_{2}\frac{\partial }{\partial x_{3}},\;\Lambda_{7}=\tau \frac{\partial }{\partial U},and\;G^{1}=-U.$$

#### 4.1.2 Conserved vectors

For Λ_1_, *δ*_1_ = 1, *δ*_2_ = *δ*_3_ = *δ*_4_ = Ψ = 0 and $G_{1}=G_{2}=G_{3}=G_{4}=0$. Then by Eq (68) we get

$$T^{\tau }=G_{1}-L\delta_{4}-\left(\Psi -U_{x_{1}}\delta_{1}-U_{x_{2}}\delta_{2}-U_{x_{3}}\delta_{3}-U_{\tau }\delta_{4}\right)\frac{\partial L}{\partial U_{\tau }}\cdot$$

This leads to

$$T^{\tau }=-U_{x_{1}}U_{\tau }$$

$$T^{x_{1}}=G_{2}-L\delta_{1}-\left(\Psi -U_{x_{1}}\delta_{1}-U_{x_{2}}\delta_{2}-U_{x_{3}}\delta_{3}-U_{\tau }\delta_{4}\right)\frac{\partial L}{\partial U_{x_{1}}},$$

simplifying the above equation, we get

$$T^{x_{1}}=\frac{A}{2}U_{x_{1}}^{2}+\frac{C}{3}U_{x_{1}}^{3}+\frac{U_{\tau }^{2}}{2}-\frac{B}{2}U_{x_{2}}^{2}-\frac{B}{2}U_{x_{3}}^{2},$$

$$T^{x_{2}}=G_{3}-L\delta_{2}-\left(\Psi -U_{x_{1}}\delta_{1}-U_{x_{2}}\delta_{2}-U_{x_{3}}\delta_{3}-U_{\tau }\delta_{4}\right)\frac{\partial L}{\partial U_{x_{2}}},$$

this leads to

$$T^{x_{2}}=BU_{x_{1}}U_{x_{2}}+DU_{x_{1}}^{2}U_{x_{2}}$$

$$T^{x_{3}}=G_{4}-L\delta_{3}-\left(\Psi -U_{x_{1}}\delta_{1}-U_{x_{2}}\delta_{2}-U_{x_{3}}\delta_{3}-U_{\tau }\delta_{4}\right)\frac{\partial L}{\partial U_{x_{3}}},$$

we obtain

$$T^{x_{3}}=BU_{x_{1}}U_{x_{3}}+DU_{x_{1}}^{2}U_{x_{3}}\cdot$$

For Λ_2_, *δ*_2_ = 1, *δ*_1_ = *δ*_3_ = *δ*_4_ = Ψ = 0 and $G_{1}=G_{2}=G_{3}=G_{4}=0$. The conserved vector associated with Λ_2_ represents the conserved quantities associated with ether symmetry Λ_2_ of the equation. These conserved quantities remain constant over time due to the symmetry provided by Λ_2_ and follow from Eq (68)

$$\begin{array}{c} \begin{array}{cc} & T^{\tau }=-U_{x_{2}}U_{\tau },\\ & T^{x_{1}}=AU_{x_{1}}U_{x_{2}}+\frac{C}{2}U_{x_{1}}^{2}U_{x_{2}}+\frac{D}{2}\left(U_{x_{2}}^{3}+U_{x_{2}}U_{x_{3}}^{2}\right),\\ & T^{x_{2}}=-\frac{A}{2}U_{x_{1}}^{2}-\frac{C}{6}U_{x_{1}}^{3}+\frac{U_{\tau }^{2}}{2}+\frac{B}{2}\left(U_{x_{2}}^{2}-U_{x_{3}}^{2}\right)+\frac{D}{2}\left(U_{x_{1}}U_{x_{2}}^{2}-U_{x_{1}}U_{x_{3}}^{2}\right),\\ & T^{x_{3}}=U_{x_{2}}\left(BU_{x_{3}}+DU_{x_{1}}U_{x_{3}}\right). \end{array} \end{array}$$ (74)

For Λ_3_, *δ*_3_ = 1, *δ*_1_ = *δ*_2_ = *δ*_4_ = Ψ = 0 and $G_{1}=G_{2}=G_{3}=G_{4}=0$. From Eq (68), the conserved vector associated with Λ_3_ becomes

$$\begin{array}{c} \begin{array}{cc} & T^{\tau }=-U_{x_{3}}U_{\tau },\\ & T^{x_{1}}=AU_{x_{1}}U_{x_{3}}+\frac{C}{2}U_{x_{1}}^{2}U_{x_{3}}+\frac{D}{2}\left(U_{x_{2}}^{2}U_{x_{3}}+U_{x_{3}}^{3}\right),\\ & T^{x_{2}}=U_{x_{2}}\left(BU_{x_{3}}+DU_{x_{1}}U_{x_{3}}\right),\\ & T^{x_{3}}=-\frac{A}{2}U_{x_{1}}^{2}-\frac{C}{6}U_{x_{1}}^{3}+\frac{U_{\tau }^{2}}{2}+\frac{B}{2}\left(-U_{x_{2}}^{2}+U_{x_{3}}^{2}\right)+\frac{D}{2}\left(-U_{x_{1}}U_{x_{2}}^{2}+U_{x_{1}}U_{x_{3}}^{2}\right). \end{array} \end{array}$$ (75)

For Λ_4_, we consider *δ*_4_ = 1, *δ*_1_ = *δ*_2_ = *δ*_3_ = Ψ = 0 and $G_{1}=G_{2}=G_{3}=G_{4}=0$. From Eq (68), the conserved vector associated with Λ_4_ becomes

$$\begin{array}{c} \begin{array}{cc} & T^{\tau }=-\frac{A}{2}U_{x_{1}}^{2}-\frac{C}{6}U_{x_{1}}^{3}-\frac{U_{\tau }^{2}}{2}-\frac{B}{2}\left(U_{x_{2}}^{2}+U_{x_{3}}^{2}\right)-\frac{D}{2}\left(U_{x_{1}}U_{x_{2}}^{2}+U_{x_{1}}U_{x_{3}}^{2}\right),\\ & T^{x_{1}}=AU_{x_{1}}U_{\tau }+\frac{C}{2}U_{x_{1}}^{2}U_{\tau }+\frac{D}{2}\left(U_{x_{2}}^{2}U_{\tau }+U_{x_{3}}^{2}U_{\tau }\right),\\ & T^{x_{2}}=U_{x_{2}}\left(BU_{\tau }+DU_{x_{1}}U_{\tau }\right),\\ & T^{x_{3}}=U_{x_{3}}\left(BU_{\tau }+DU_{x_{1}}U_{\tau }\right). \end{array} \end{array}$$ (76)

For Λ_5_, we follow Ψ = 1, *δ*_1_ = *δ*_2_ = *δ*_3_ = *δ*_4_ = 0 and $G_{1}=G_{2}=G_{3}=G_{4}=0$. From Eq (68), the conserved vector associated with Λ_5_ becomes

$$\begin{array}{c} \begin{array}{cc} & T^{\tau }=U_{\tau },\\ & T^{x_{1}}=-AU_{x_{1}}-\frac{C}{2}U_{x_{1}}^{2}-\frac{D}{2}\left(U_{x_{2}}^{2}+U_{x_{3}}^{2}\right),\\ & T^{x_{2}}=-\left(BU_{x_{2}}+DU_{x_{1}}U_{x_{2}}\right),\\ & T^{x_{3}}=-\left(BU_{x_{3}}+DU_{x_{1}}U_{x_{3}}\right). \end{array} \end{array}$$ (77)

For Λ_6_, we have *δ*_2_ = *x*_3_, *δ*_3_ = −*x*_2_, *δ*_1_ = *δ*_4_ = 0 and $G_{1}=G_{2}=G_{3}=G_{4}=0$. From Eq (68), the conserved vector associated with Λ_6_ becomes

$$\begin{array}{c} \begin{array}{cc} & T^{\tau }=\left(-x_{3}U_{x_{2}}+x_{2}U_{x_{3}}\right)U_{\tau },\\ & T^{x_{1}}=\left(AU_{x_{1}}+\frac{C}{2}U_{x_{1}}^{2}+\frac{D}{2}\left(U_{x_{2}}^{2}+U_{x_{3}}^{2}\right)\right)\left(x_{3}U_{x_{2}}-x_{2}U_{x_{3}}\right),\\ & T^{x_{2}}=-x_{3}\left(\frac{A}{2}U_{x_{1}}^{2}+\frac{C}{6}U_{x_{1}}^{3}-\frac{U_{\tau }^{2}}{2}-\frac{B}{2}\left(U_{x_{2}}^{2}-U_{x_{3}}^{2}\right)-\frac{D}{2}\left(U_{x_{1}}U_{x_{2}}^{2}-U_{x_{1}}U_{x_{3}}^{2}\right)\right)-yU_{x_{2}}U_{x_{3}}\left(B+DU_{x_{1}}\right),\\ & T^{x_{3}}=x_{2}\left(\frac{A}{2}U_{x_{1}}^{2}+\frac{C}{6}U_{x_{1}}^{3}-\frac{U_{\tau }^{2}}{2}+\frac{B}{2}\left(U_{x_{2}}^{2}-U_{x_{3}}^{2}\right)+\frac{D}{2}\left(U_{x_{1}}U_{x_{2}}^{2}-U_{x_{1}}U_{x_{3}}^{2}\right)\right)+x_{3}U_{x_{2}}U_{x_{3}}\left(B+DU_{x_{1}}\right). \end{array} \end{array}$$ (78)

For Λ_7_, we have Ψ = *τ*, *δ*_1_ = *δ*_2_ = *δ*_3_ = *δ*_4_ = 0 and $G_{1}=-U,G_{2}=G_{3}=G_{4}=0$. From Eq (68), the conserved vector associated with Λ_7_ becomes

$$\begin{array}{c} \begin{array}{cc} & T^{\tau }=-U+\tau U_{\tau },\\ & T^{x_{1}}=-\tau \left(AU_{x_{1}}+\frac{C}{2}U_{x_{1}}^{2}+\frac{D}{2}\left(U_{x_{2}}^{2}+U_{x_{3}}^{2}\right)\right),\\ & T^{x_{2}}=-\tau \left(BU_{x_{2}}+DU_{x_{1}}U_{x_{2}}\right),\\ & T^{x_{3}}=-\tau \left(BU_{x_{3}}+DU_{x_{1}}U_{x_{3}}\right). \end{array} \end{array}$$ (79)

## 5 Damped elastic wave equation

The addition of the damping term *U*_*τ*_ to the non-linear elastic wave Eq (13) transforms it into a non-linear damped elastic wave equation given by

$$\begin{array}{c} \begin{array}{c} U_{\tau \tau }-AU_{x_{1}x_{1}}-B\left(U_{x_{2}x_{2}}+U_{x_{3}x_{3}}\right)-CU_{x_{1}}U_{x_{1}x_{1}}-D\left(U_{x_{1}}U_{x_{2}x_{2}}+U_{x_{1}}U_{x_{3}x_{3}}\right)\\ -2D\left(U_{x_{2}}U_{x_{1}x_{2}}+U_{x_{3}}U_{x_{1}x_{3}}\right)+\gamma U_{\tau }=0, \end{array} \end{array}$$ (80)

where *γ* is the damping parameter.

In this section, we present a comprehensive Lie symmetry analysis of the nonlinear damped elastic wave Eq (80).

We assume the following form of the vector field

$$\Lambda =\delta_{1}\frac{\partial }{\partial x_{1}}+\delta_{2}\frac{\partial }{\partial x_{2}}+\delta_{3}\frac{\partial }{\partial x_{3}}+\delta_{4}\frac{\partial }{\partial \tau }+\Psi \frac{\partial }{\partial U}\cdot$$

The invariance condition for the Eq (80) is

$$\begin{array}{c} \begin{array}{cc} & \Lambda^{\left[2\right]}(U_{\tau \tau }-AU_{x_{1}x_{1}}-B\left(U_{x_{2}x_{2}}+U_{x_{3}x_{3}}\right)-CU_{x_{1}}U_{x_{1}x_{1}}-D\left(U_{x_{1}}U_{x_{2}x_{2}}+U_{x_{1}}U_{x_{3}x_{3}}\right)\\ & -2D\left(U_{x_{2}}U_{x_{1}x_{2}}+U_{x_{3}}U_{x_{1}x_{3}}\right)+\gamma U_{\tau }{)|}_{\left(6.1\right)}=0. \end{array} \end{array}$$ (81)

Upon comparing the coefficients of derivatives of *U* in the given expressions, we get a set of determining system and solving it, we obtain

$$\begin{array}{c} \begin{array}{cc} & \delta_{1_{x_{1}}}=\delta_{1_{x_{2}}}=\delta_{1_{x_{3}}}=\delta_{1_{\tau }}=\delta_{1_{U}}=0,\\ & \delta_{4_{x_{1}}}=\delta_{4_{x_{2}}}=\delta_{4_{x_{3}}}=\delta_{4_{\tau }}=\delta_{4_{U}}=0,\\ & \delta_{2_{x_{1}}}=\delta_{2_{x_{2}}}=\delta_{2_{\tau }}=\delta_{2_{U}}=0,\\ & \delta_{3_{x_{1}}}=\delta_{3_{x_{3}}}=\delta_{3_{\tau }}=\delta_{3_{U}}=0,\\ & \Psi_{x_{1}}=\Psi_{x_{2}}=\Psi_{x_{3}}=\Psi_{U}=0,\\ & \delta_{3_{x_{2}}}+\delta_{2_{x_{3}}}=0,\\ & \Psi_{\tau \tau }+\gamma \Psi_{\tau }=0,\\ & \delta_{2_{x_{3}x_{3}}}=0, \end{array} \end{array}$$ (82)

The infinitesimals we obtain here are given by

$$\delta_{1}=C_{1},\;\delta_{2}=C_{2}x_{3}+C_{3},\;\delta_{3}=-C_{2}x_{2}+C_{4},\;\delta_{4}=C_{5},\;\Psi =C_{6}+C_{7}e^{-\gamma \tau }.$$

We follow the following symmetry algebra given by

$$\begin{array}{c} \Lambda_{1}=\frac{\partial }{\partial \tau },\;\Lambda_{2}=\frac{\partial }{\partial U},\;\Lambda_{3}=\frac{\partial }{\partial x_{1}},\;\Lambda_{4}=\frac{\partial }{\partial x_{2}},\;\Lambda_{5}=\frac{\partial }{\partial x_{3}},\\ \Lambda_{6}=e^{-\gamma \tau }\frac{\partial }{\partial U},\;\Lambda_{7}=x_{3}\frac{\partial }{\partial x_{2}}-x_{2}\frac{\partial }{\partial x_{3}}\cdot \end{array}$$

Table 4 lists the commutator relation for the generators of symmetry algebra (5).

**Table 4:** Commutator table.

[Λ_*i*_, Λ_*j*_]	Λ_1_	Λ_2_	Λ_3_	Λ_4_	Λ_5_	Λ_6_	Λ_7_
Λ_1_	0	0	0	0	0	−*γ*Λ_6_	0
Λ_2_	0	0	0	0	0	0	0
Λ_3_	0	0	0	0	0	0	0
Λ_4_	0	0	0	0	0	0	- Λ_5_
Λ_5_	0	0	0	0	0	0	Λ_4_
Λ_6_	*γ*Λ_6_	0	0	0	0	0	0
Λ_7_	0	0	0	Λ_5_	−Λ_4_	0	0

Following a similar procedure, we obtain the optimal system of symmetry algebra of Eq (80) given by,

$$\begin{array}{cc} \begin{array}{l} \begin{array}{c} R_{1}=\Lambda_{1} \end{array}\\ \begin{array}{c} R_{2}=\Lambda_{1}+\kappa \Lambda_{2},\;\kappa \neq 0 \end{array}\\ \begin{array}{c} R_{3}=\Lambda_{1}+\kappa \Lambda_{3},\;\kappa \neq 0 \end{array}\\ \begin{array}{c} R_{4}=\Lambda_{2} \end{array}\\ \begin{array}{c} R_{5}=\Lambda_{3} \end{array}\\ \begin{array}{c} R_{6}=\Lambda_{1}+\kappa \Lambda_{2}+\sigma \Lambda_{3},\;\kappa ,\sigma \neq 0 \end{array}\\ \begin{array}{c} R_{7}=\Lambda_{1}+\kappa \Lambda_{2}+\sigma \Lambda_{4},\;\kappa ,\sigma \neq 0 \end{array}\\ \begin{array}{c} R_{8}=\Lambda_{1}+\kappa \Lambda_{4},\;\kappa \neq 0 \end{array}\\ \begin{array}{c} R_{9}=\Lambda_{1}+\kappa \Lambda_{5},\;\kappa \neq 0 \end{array}\\ \begin{array}{c} R_{10}=\Lambda_{1}+\kappa \Lambda_{7},\;\kappa \neq 0 \end{array}\\ \begin{array}{c} R_{11}=\Lambda_{4} \end{array}\\ \begin{array}{c} R_{12}=\Lambda_{5} \end{array}\\ \begin{array}{c} R_{13}=\Lambda_{1}+\Lambda_{2}+\kappa \Lambda_{3}+\sigma \Lambda_{4},\;\kappa ,\sigma \neq 0 \end{array}\\ \begin{array}{c} R_{14}=\Lambda_{2}+\kappa \Lambda_{3},\;\kappa \neq 0 \end{array}\\ \begin{array}{c} R_{15}=\Lambda_{2}+\kappa \Lambda_{4},\;\kappa \neq 0 \end{array}\\ \begin{array}{c} R_{16}=\Lambda_{2}+\kappa \Lambda_{5},\;\kappa \neq 0 \end{array}\\ \begin{array}{c} R_{17}=\Lambda_{2}+\kappa \Lambda_{6},\;\kappa \neq 0 \end{array}\\ \begin{array}{c} R_{18}=\Lambda_{2}+\kappa \Lambda_{7},\;\kappa \neq 0 \end{array} \end{array} & \begin{array}{l} \begin{array}{c} R_{19}=\Lambda_{3}+\kappa \Lambda_{4},\;\kappa \neq 0 \end{array}\\ \begin{array}{c} R_{20}=\Lambda_{3}+\kappa \Lambda_{5},\;\kappa \neq 0 \end{array}\\ \begin{array}{c} R_{21}=\Lambda \pm \Lambda_{6} \end{array}\\ \begin{array}{c} R_{22}=\Lambda_{6} \end{array}\\ \begin{array}{c} R_{23}=\Lambda_{1}+\kappa \Lambda_{3}+\sigma \Lambda_{4},\;\kappa ,\sigma \neq 0 \end{array}\\ \begin{array}{c} R_{24}=\Lambda_{7} \end{array}\\ \begin{array}{c} R_{25}=\Lambda_{1}+\Lambda_{2}+\kappa \Lambda_{3}+\sigma \Lambda_{5},\;\kappa ,\sigma \neq 0 \end{array}\\ \begin{array}{c} R_{26}=\Lambda_{1}+\Lambda_{2}+\kappa \Lambda_{3}+\sigma \Lambda_{7},\;\kappa ,\sigma \neq 0 \end{array}\\ \begin{array}{c} R_{27}=\Lambda_{4}\pm \Lambda_{6} \end{array}\\ \begin{array}{c} R_{28}=\Lambda_{2}+\Lambda_{3}+\kappa \Lambda_{5}+\sigma \Lambda_{7},\;\kappa ,\sigma \neq 0 \end{array}\\ \begin{array}{c} R_{29}=\Lambda_{1}+\kappa \Lambda_{3}+\sigma \Lambda_{5},\;\kappa ,\sigma \neq 0 \end{array}\\ \begin{array}{c} R_{30}=\Lambda_{1}+\kappa \Lambda_{2}+\sigma \Lambda_{5},\;\kappa ,\sigma \neq 0 \end{array}\\ \begin{array}{c} R_{31}=\Lambda_{1}+\kappa \Lambda_{2}+\sigma \Lambda_{7},\;\kappa ,\sigma \neq 0 \end{array}\\ \begin{array}{c} R_{32}=\Lambda_{3}+\kappa \Lambda_{7},\;\kappa \neq 0 \end{array}\\ \begin{array}{c} R_{33}=\Lambda_{5}\pm \Lambda_{6} \end{array}\\ \begin{array}{c} R_{34}=\Lambda_{6}\pm \Lambda_{7} \end{array}\\ \begin{array}{c} R_{35}=\Lambda_{1}+\kappa \Lambda_{3}+\sigma \Lambda_{7},\;\kappa ,\sigma \neq 0, \end{array} \end{array} \end{array}$$

## 6 Group invariant solutions

**Invariant Solutions by Using** $R_{1}=\Lambda_{1}.$

First, let us consider the infinitesimal generator $\Lambda_{1}=\frac{\partial }{\partial \tau }\cdot$

The characteristic equation associated with Λ_1_ is given by

$$\frac{dx_{1}}{0}=\frac{dx_{2}}{0}=\frac{dx_{3}}{0}=\frac{d\tau }{1}=\frac{dU}{0}\cdot$$

From the characteristic equation, we can deduce that *x*_1_ = *α*, *x*_2_ = *β*, *x*_3_ = λ, *U* = *X*(*α*, *β*, λ). Using these invariant variables, (80) is transformed into the equation given by

$$\begin{array}{c} \left(-CX_{\alpha }-A\right)X_{\alpha \alpha }+\left(-DX_{\alpha }-B\right)X_{\beta \beta }+\left(-DX_{\alpha }-B\right)X_{\lambda \lambda }-2D\left(X_{\beta }X_{\alpha \beta }+X_{\lambda }X_{\alpha \lambda }\right)=0. \end{array}$$ (83)

Infinitesimals of (83) are written as

$$\begin{array}{c} \begin{array}{cc} \delta_{\alpha } & =c_{2}\alpha +c_{5},\\ \delta_{\beta } & =-c_{1}\lambda +c_{2}\beta +c_{6},\\ \delta_{\lambda } & =c_{1}\beta +c_{2}\lambda +c_{3},\\ \Psi_{X} & =c_{2}X+c_{4}. \end{array} \end{array}$$ (84)

**Case 1:** By taking *c*_5_ = 1, *c*_6_ = 1 and all other constants zero, the characteristic equation associated with (84) is

$$\frac{d\alpha }{1}=\frac{d\beta }{1}=\frac{d\lambda }{0}=\frac{dX}{0},$$

which implies that λ = *r*, *β* − *α* = *s* and *X*(*α*, *β*, λ) = *Y*(*r*, *s*). Using these invariant variables, (83) is transformed into the following equation

$$\begin{array}{c} \left(\left(C+3D\right)Y_{s}-A-B\right)Y_{ss}+\left(DY_{s}-B\right)Y_{rr}+2DY_{r}Y_{rs}=0. \end{array}$$ (85)

Infinitesimals of Eq (85) becomes

$$\begin{array}{c} \begin{array}{cc} \delta_{r} & =c_{1}r+c_{2},\\ \delta_{s} & =c_{1}s+c_{4},\\ \Psi_{Y} & =c_{1}Y+c_{3}. \end{array} \end{array}$$ (86)

**Case 1a:** By taking *c*_3_ = 1, *c*_4_ = 1 and all other constants zero, the characteristic equation associated with (86) is

$$\frac{dr}{0}=\frac{ds}{1}=\frac{dY}{1},$$

which implies that *r* = *p* and *Y*(*r*, *s*) = *s* + *Z*(*p*). Using these invariant variables, (85) is transformed into the following ODE

$$\begin{array}{c} -Z^{\prime \prime }\left(B-D\right)=0. \end{array}$$ (87)

This implies,

$$\begin{array}{c} Z\left(p\right)=c_{1}p+c_{2}, \end{array}$$ (88)

which provides,

$$\begin{array}{c} Y\left(r,s\right)=c_{1}r+c_{2}+s, \end{array}$$ (89)

this implies,

$$\begin{array}{c} X\left(\alpha ,\beta ,\lambda \right)=c_{1}\lambda +c_{2}+\left(\beta -\alpha \right). \end{array}$$ (90)

So, the solution of (80) in original variables is

$$\begin{array}{c} U\left(x_{1},x_{2},x_{3},\tau \right)=c_{1}x_{3}+c_{2}+\left(x_{2}-x_{1}\right). \end{array}$$ (91)

**Invariant Solutions by Using** $R_{5}=\Lambda_{3}.$

First, let us consider the infinitesimal generator $\Lambda_{3}=\frac{\partial }{\partial x_{1}}\cdot$

The characteristic equation associated with Λ_3_ is given by

$$\frac{dx_{1}}{1}=\frac{dx_{2}}{0}=\frac{dx_{3}}{0}=\frac{d\tau }{0}=\frac{dU}{0}\cdot$$

From the characteristic equation, we can deduce that *τ* = *α*, *x*_2_ = *β*, *x*_3_ = λ, *U* = *X*(*α*, *β*, λ). Using these invariant variables, (80) is transformed into the equation given by

$$\begin{array}{c} X_{\alpha \alpha }-BX_{\beta \beta }-BX_{\lambda \lambda }+\gamma X_{\alpha }=0. \end{array}$$ (92)

Infinitesimals of (92) are written as

$$\begin{array}{c} \begin{array}{cc} \delta_{\alpha } & =\frac{c_{2}}{B}\lambda -\frac{2}{\gamma }c_{4}\beta +c_{13},\\ \delta_{\beta } & =-c_{1}\lambda -\frac{2}{\gamma }c_{4}B\alpha +c_{12},\\ \delta_{\lambda } & =c_{1}\beta +c_{2}\alpha +c_{3},\\ \Psi_{X} & =\frac{1}{e^{\sqrt{a_{2}}\beta }}(2(c_{10}\;\text{sin}\;(\frac{\sqrt{a_{2}B-a_{1}}\lambda }{\sqrt{B}})+c_{11}\;\text{cos}\;(\frac{\sqrt{a_{2}B-a_{1}}\lambda }{\sqrt{B}}))(e^{-\frac{\alpha \sqrt{\gamma^{2}+4a_{1}}}{2}}c_{7}\\ & +e^{\frac{\alpha \sqrt{\gamma^{2}+4a_{1}}}{2}}c_{6})B({\left(e^{\sqrt{a_{2}}\beta }\right)}^{2}c_{8}+c_{9})e^{-\frac{\alpha \gamma }{2}}+2e^{\sqrt{a_{2}}\beta }(\left(c_{4}\beta +c_{5}\right)B-\frac{c_{2}}{2}\gamma \lambda )X). \end{array} \end{array}$$ (93)

**Case 2:** By taking *c*_3_ = 1 and all other constants zero, the characteristic equation associated with (93) is

$$\frac{d\alpha }{0}=\frac{d\beta }{0}=\frac{d\lambda }{1}=\frac{dX}{0},$$

which implies that *α* = *r*, *β* = *s*, and *X*(*α*, *β*, λ) = *Y*(*r*, *s*). Using these invariant variables, (92) is transformed into the following equation

$$\begin{array}{c} Y_{rr}-BY_{ss}+\gamma Y_{r}=0. \end{array}$$ (94)

Infinitesimals of Eq (94) becomes

$$\begin{array}{c} \begin{array}{cc} \delta_{r} & =-\frac{2}{\gamma }c_{1}s+c_{5},\\ \delta_{s} & =-\frac{2}{\gamma }Bc_{1}r+c_{6},\\ \Psi_{Y} & =\left(c_{1}s+c_{2}\right)Y+c_{3}\frac{{\left(e^{\frac{-a_{1}^{2}\sqrt{B}\left(\sqrt{B}r+s\right)}{4\sqrt{B}a_{1}+\gamma }}\right)}^{4}e^{\frac{a_{1}\gamma \left(\sqrt{B}r+s\right)}{4\sqrt{B}a_{1}+\gamma }}}{{\left(e^{\frac{\gamma a_{1}\sqrt{B}\left(-\frac{s}{\sqrt{B}}+r\right)}{8\sqrt{B}a_{1}+2\gamma }}\right)}^{2}}c_{4}. \end{array} \end{array}$$ (95)

**Case 2a:** By taking *c*_5_ = 1, *c*_6_ = 1 and all other constants zero, the characteristic equation associated with (95) is

$$\frac{dr}{1}=\frac{ds}{1}=\frac{dY}{0},$$

which implies that −*r* + *s* = *p* and *Y*(*r*, *s*) = *s* + *Z*(*p*). Using these invariant variables, (94) is transformed into the following ODE

$$\begin{array}{c} Z^{\prime \prime }-BZ^{\prime \prime }-\gamma Z^{\prime }=0. \end{array}$$ (96)

This implies,

$$\begin{array}{c} Z\left(p\right)=c_{1}+c_{2}e^{\frac{-\gamma p}{B-1}}, \end{array}$$ (97)

which provides,

$$\begin{array}{c} Y\left(r,s\right)=c_{1}+c_{2}e^{\frac{-\gamma (s-r)}{B-1}}, \end{array}$$ (98)

this implies,

$$\begin{array}{c} X\left(\alpha ,\beta ,\lambda \right)=c_{1}+c_{2}e^{\frac{-\gamma (\beta -\alpha )}{B-1}}. \end{array}$$ (99)

So, the solution of (80) in original variables is

$$\begin{array}{c} U\left(x_{1},x_{2},x_{3},\tau \right)=c_{1}+c_{2}e^{\frac{-\gamma (x_{2}-\tau )}{B-1}}. \end{array}$$ (100)

**Invariant Solutions by Using** $R_{12}=\Lambda_{5}.$

First, let us consider the infinitesimal generator $\Lambda_{5}=\frac{\partial }{\partial x_{3}}\cdot$

The characteristic equation associated with Λ_5_ is given by

$$\frac{dx_{1}}{0}=\frac{dx_{2}}{0}=\frac{dx_{3}}{1}=\frac{d\tau }{0}=\frac{dU}{0}\cdot$$

From the characteristic equation, we can deduce that *τ* = *α*, *x*_1_ = *β*, *x*_2_ = λ, *U* = *X*(*α*, *β*, λ). Using these invariant variables, (80) is transformed into the equation given by

$$\begin{array}{c} \left(-CX_{\beta }-A\right)X_{\beta \beta }+\left(-DX_{\beta }-B\right)X_{\lambda \lambda }-2DX_{\lambda }X_{\beta \lambda }+\gamma X_{\alpha }+X_{\alpha \alpha }=0. \end{array}$$ (101)

Infinitesimals of (101) are written as

$$\begin{array}{c} \begin{array}{cc} \delta_{\alpha } & =c_{3},\\ \delta_{\beta } & =c_{4},\\ \delta_{\lambda } & =c_{5},\\ \Psi_{X} & =c_{1}+c_{2}e^{-\alpha \gamma }. \end{array} \end{array}$$ (102)

**Case 3:** By taking *c*_2_ = 1, *c*_5_ = 1 and all other constants zero, the characteristic equation associated with (102) is

$$\frac{d\alpha }{0}=\frac{d\beta }{0}=\frac{d\lambda }{1}=\frac{dX}{e^{-\alpha \gamma }},$$

which implies that *α* = *r*, *β* = *s*, and *X*(*α*, *β*, λ) = λ*e*^−*αγ*^ + *Y*(*r*, *s*). Using these invariant variables, (101) is transformed into the following equation

$$\begin{array}{c} \left(-CY_{s}-A\right)Y_{ss}+\gamma Y_{r}+Y_{rr}=0. \end{array}$$ (103)

Infinitesimals of Eq (94) becomes

$$\begin{array}{c} \begin{array}{cc} \delta_{r} & =c_{3},\\ \delta_{s} & =c_{1}s+c_{2},\\ \Psi_{Y} & =3c_{1}Y+\frac{2A}{C}c_{1}s+c_{4}+c_{5}e^{-\gamma r}. \end{array} \end{array}$$ (104)

**Case 3a:** By taking *c*_2_ = 1, *c*_5_ = 1 and all other constants zero, the characteristic equation associated with (104) is

$$\frac{dr}{0}=\frac{ds}{1}=\frac{dY}{e^{-\gamma r}},$$

which implies that *r* = *p* and *Y*(*r*, *s*) = *se*^−*γr*^ + *Z*(*p*). Using these invariant variables, (103) is transformed into the following ODE

$$\begin{array}{c} Z^{\prime \prime }+\gamma Z^{\prime }=0. \end{array}$$ (105)

This implies,

$$\begin{array}{c} Z\left(p\right)=c_{1}+c_{2}e^{-\gamma p}, \end{array}$$ (106)

which provides,

$$\begin{array}{c} Y\left(r,s\right)=c_{1}+\left(c_{2}+s\right)e^{-\gamma r}, \end{array}$$ (107)

this implies,

$$\begin{array}{c} X\left(\alpha ,\beta ,\lambda \right)=c_{1}+\left(c_{2}+\beta \right)e^{-\gamma \alpha }+\lambda e^{-\alpha \gamma }, \end{array}$$ (108)

So, the solution of (80) in original variables is

$$\begin{array}{c} U\left(x_{1},x_{2},x_{3},\tau \right)=c_{1}+\left(c_{2}+x_{1}\right)e^{-\gamma \tau }+x_{2}e^{-x_{1}\gamma }. \end{array}$$ (109)

**Invariant Solutions by Using** $R_{15}=\Lambda_{2}+\kappa \Lambda_{4}.$

First, let us consider the infinitesimal generator $\Lambda_{2}+\kappa \Lambda_{4}=\frac{\partial }{\partial U}+\kappa \frac{\partial }{\partial x_{2}}\cdot$

The characteristic equation associated with Λ_2_ + *κ*Λ_4_ is given by

$$\frac{dx_{1}}{0}=\frac{dx_{2}}{\kappa }=\frac{dx_{3}}{0}=\frac{d\tau }{0}=\frac{dU}{1}\cdot$$

From the characteristic equation, we can deduce that $\tau =\alpha ,\;x_{1}=\beta ,\;x_{3}=\lambda ,\;U=\frac{y}{\kappa }+X\left(\alpha ,\beta ,\lambda \right)$. Using these invariant variables, (80) is transformed into the equation given by

$$\begin{array}{c} \left(-CX_{\beta }-A\right)X_{\beta \beta }+\left(-DX_{\beta }-B\right)X_{\lambda \lambda }-2DX_{\lambda }X_{\beta \lambda }+\gamma X_{\alpha }+X_{\alpha \alpha }=0. \end{array}$$ (110)

Infinitesimals of (110) are written as

$$\begin{array}{c} \begin{array}{cc} \delta_{\alpha } & =c_{3},\\ \delta_{\beta } & =c_{4},\\ \delta_{\lambda } & =c_{5},\\ \Psi_{X} & =c_{1}+c_{2}e^{-\alpha \gamma }. \end{array} \end{array}$$ (111)

**Case 4:** By taking *c*_3_ = 1, *c*_4_ = 1 and all other constants zero, the characteristic equation associated with (111) is

$$\frac{d\alpha }{1}=\frac{d\beta }{1}=\frac{d\lambda }{0}=\frac{dX}{0},$$

which implies that λ = *r*, −*α* + *β* = *s* and *X*(*α*, *β*, λ) = *Y*(*r*, *s*). Using these invariant variables, (110) is transformed into the following equation

$$\begin{array}{c} \left(-CY_{s}-A+1\right)Y_{ss}+\left(-DY_{s}-B\right)Y_{rr}-2DY_{r}Y_{rs}-\gamma Y_{s}=0. \end{array}$$ (112)

Infinitesimals of Eq (112) becomes

$$\begin{array}{c} \begin{array}{cc} \delta_{r} & =c_{2},\\ \delta_{s} & =c_{3},\\ \Psi_{Y} & =c_{1}. \end{array} \end{array}$$ (113)

**Case 4a:** By taking *c*_1_ = 1, *c*_3_ = 1 and all other constants zero, the characteristic equation associated with (113) is

$$\frac{dr}{0}=\frac{ds}{1}=\frac{dY}{1},$$

which implies that *r* = *p*, *Y*(*r*, *s*) = *s* + *Z*(*p*). Using these invariant variables, (112) is transformed into the following ODE

$$\begin{array}{c} \left(-B-D\right)Z^{\prime \prime }-\gamma =0. \end{array}$$ (114)

This implies,

$$\begin{array}{c} Z\left(p\right)=\frac{-\gamma }{2(B+D)}p^{2}+c_{1}p+c_{2}, \end{array}$$ (115)

which provides,

$$\begin{array}{c} Y\left(r,s\right)=\frac{-\gamma }{2(B+D)}r^{2}+c_{1}r+c_{2}+s, \end{array}$$ (116)

this implies,

$$\begin{array}{c} X\left(\alpha ,\beta ,\lambda \right)=\frac{-\gamma }{2(B+D)}\lambda^{2}+c_{1}\lambda +c_{2}+\left(\beta -\alpha \right), \end{array}$$ (117)

So, the solution of (80) in original variables is

$$\begin{array}{c} U\left(x_{1},x_{2},x_{3},\tau \right)=\frac{y}{\kappa }+\frac{-\gamma }{2(B+D)}x_{3}^{2}+c_{1}x_{3}+c_{2}+\left(x_{1}-\tau \right). \end{array}$$ (118)

**Invariant Solutions by Using** $R_{6}=\Lambda_{1}+\kappa \Lambda_{2}+\sigma \Lambda_{3}.$

First, let us consider the infinitesimal generator $\Lambda_{1}+\kappa \Lambda_{2}+\sigma \Lambda_{3}=\frac{\partial }{\partial \tau }+\kappa \frac{\partial }{\partial U}+\sigma \frac{\partial }{\partial x_{1}}\cdot$

The characteristic equation associated with Λ_1_ + *κ*Λ_2_ + *σ*Λ_3_ is given by

$$\frac{dx_{1}}{\sigma }=\frac{dx_{2}}{0}=\frac{dx_{3}}{0}=\frac{d\tau }{1}=\frac{dU}{\kappa }\cdot$$

From the characteristic equation, we can deduce that $x_{2}=\alpha ,\;x_{3}=\beta ,\;\tau -\frac{x_{1}}{\sigma }=\lambda ,\;U=\frac{\kappa }{\sigma }x_{1}+X\left(\alpha ,\beta ,\lambda \right)$. Using these invariant variables, (80) is transformed into the equation given by

$$\begin{array}{c} \begin{array}{c} \left(\sigma^{3}-A\sigma +CX_{\lambda }-C\kappa \right)X_{\lambda \lambda }+\sigma^{2}(\left(-B\sigma +DX_{\lambda }-\kappa D\right)X_{\beta \beta }+\left(-B\sigma +DX_{\lambda }-\kappa D\right)X_{\alpha \alpha }\\ +\sigma \gamma X_{\lambda }+2DX_{\alpha }X_{\alpha \lambda }+2DX_{\beta }X_{\beta \lambda })=0, \end{array} \end{array}$$ (119)

Infinitesimals of (119) are written as

$$\begin{array}{c} \begin{array}{cc} \delta_{\alpha } & =c_{1}\beta +c_{2},\\ \delta_{\beta } & =-c_{1}\alpha +c_{5},\\ \delta_{\lambda } & =c_{4},\\ \Psi_{X} & =c_{3}. \end{array} \end{array}$$ (120)

**Case 5:** By taking *c*_3_ = 1, *c*_5_ = 1 and all other constants zero, the characteristic equation associated with (120) is

$$\frac{d\alpha }{0}=\frac{d\beta }{1}=\frac{d\lambda }{0}=\frac{dX}{1},$$

which implies that *α* = *r*, λ = *s*, and *X*(*α*, *β*, λ) = *β* + *Y*(*r*, *s*). Using these invariant variables, (119) is transformed into the following equation

$$\begin{array}{c} \left(\sigma^{3}-A\sigma +CY_{s}-\kappa C\right)Y_{ss}+2((\frac{D}{2}Y_{s}-\frac{B}{2}\sigma -\frac{D}{2}\kappa )Y_{rr}+DY_{r}Y_{rs}+\frac{\sigma }{2}\gamma Y_{s})\sigma^{2}=0. \end{array}$$ (121)

Infinitesimals of Eq (121) becomes

$$\begin{array}{c} \begin{array}{cc} \delta_{r} & =c_{2},\\ \delta_{s} & =c_{3},\\ \Psi_{Y} & =c_{1}. \end{array} \end{array}$$ (122)

**Case 5a:** By taking *c*_1_ = 1, *c*_3_ = 1 and all other constants zero, the characteristic equation associated with (113) is

$$\frac{dr}{0}=\frac{ds}{1}=\frac{dY}{1},$$

which implies that *r* = *p*, *Y*(*r*, *s*) = *s* + *Z*(*p*). Using these invariant variables, (121) is transformed into the following ODE

$$\begin{array}{c} -\left(\left(\sigma B+D\left(\kappa -1\right)\right)Z^{\prime \prime }-\gamma \sigma \right)\sigma^{2}=0, \end{array}$$ (123)

This implies,

$$\begin{array}{c} Z\left(p\right)=\frac{-\gamma \sigma }{2(\sigma B+D(\kappa -1))}p^{2}+c_{1}p+c_{2}, \end{array}$$ (124)

which provides,

$$\begin{array}{c} Y\left(r,s\right)=\frac{-\gamma \sigma }{2(\sigma B+D(\kappa -1))}r^{2}+c_{1}r+c_{2}+s, \end{array}$$ (125)

this implies,

$$\begin{array}{c} X\left(\alpha ,\beta ,\lambda \right)=\frac{-\gamma \sigma }{2(\sigma B+D(\kappa -1))}\alpha^{2}+c_{1}\alpha +c_{2}+\lambda +\beta , \end{array}$$ (126)

So, the solution of (80) in original variables is

$$\begin{array}{c} U\left(x_{1},x_{2},x_{3},\tau \right)=\frac{\kappa }{\sigma }x_{1}+\frac{-\gamma \sigma }{2(\sigma B+D(\kappa -1))}x_{2}^{2}+c_{1}x_{2}+c_{2}+\tau -\frac{x_{1}}{\sigma }+x_{3}. \end{array}$$ (127)

## 7 Partial noether approach

In cases in which the standard Lagrangian is either unavailable or challenging to ascertain, an alternative approach involves the use of a partial Lagrangian. Conservation laws are then derived using the partial Noether approach, which was introduced by Kara and Mahomed [21].

**Partial Lagrangian** The *m*^*th*^ order differential system (61) can be expressed as follows

$$\begin{array}{c} H_{\alpha }=H_{\alpha }^{0}+H_{\alpha }^{1}=0, \end{array}$$ (128)

A function $L=L(x,U,U^{\left(1\right)},\cdots ,U^{\left(n\right)}),n\leq m$, is referred to as a partial Lagrangian of the system (61) if the system (61) can be represented in the form

$$\frac{\delta L}{\delta U^{\alpha }}=f_{\alpha }^{\beta }H_{\beta }^{1}$$

subject to the condition that $H_{\beta }^{1}\neq 0$ for a certain *β*. Here, $\left(f_{\alpha }^{\beta }\right)$ denotes an invertible matrix.

**Partial Noether Symmetries** The operator Λ as defined in (62), which fulfills the condition

$$\begin{array}{c} \Lambda \left(L\right)+L\left(D_{i}\Psi^{i}\right)=D_{i}\left(G^{i}\right)+\left(\Psi^{\varpi }-\Psi^{j}U_{j}^{\varpi }\right)\frac{\delta L}{\delta U^{\varpi }},\;i=1,2,\cdots ,n,\;\varpi =1,2,\cdots ,m, \end{array}$$ (129)

is classified as a partial Noether symmetry generator, corresponding to the partial Lagrangian L.

**Conserved Vectors** To find the conserved vector related to the system described by Eq (61), which is associated with a symmetry generator (partial Noether) Λ corresponding to the partial Lagrangian L, we utilize Eq (68).

### 7.1 Partial Noether approach to Eq (80)

In this subsection, we utilize Noether’s partial approach [21] to derive the conservation laws of Eq (80). Initially, we identify the partial Noether symmetries, which are infinitesimal transformations preserving Eq (80). Subsequently, we established associated conservation laws corresponding to these symmetries. The partial Noether’s approach is a powerful method for uncovering conserved quantities. We assume the partial Lagrangian for Eq (80)

$$\begin{array}{c} L=\frac{AU_{x_{1}}^{2}}{2}+\frac{B}{2}\left(U_{x_{2}}^{2}+U_{x_{3}}^{2}\right)+\frac{CU_{x_{1}}^{3}}{6}-\frac{U_{\tau }^{2}}{2}+\frac{D}{2}\left(U_{x_{1}}U_{x_{2}}^{2}+U_{x_{1}}U_{x_{3}}^{2}\right). \end{array}$$ (130)

By using the Euler operator (65), we get

$$\frac{\delta L}{\delta U}=-\gamma U_{\tau }\cdot$$

### 7.2 Partial Noether symmetries

The vector field

$$\Lambda =\delta_{1}\frac{\partial }{\partial x_{1}}+\delta_{2}\frac{\partial }{\partial x_{2}}+\delta_{3}\frac{\partial }{\partial x_{3}}+\delta_{4}\frac{\partial }{\partial \tau }+\Psi \frac{\partial }{\partial U},$$

is referred to as a partial Noether symmetry of Eq (80) concerning the partial Lagrangian (130) if it fulfills the condition

$$\begin{array}{c} \begin{array}{cc} & \Lambda^{\left[1\right]}L+L\left(D_{\tau }\delta_{4}+D_{x_{1}}\delta_{1}+D_{x_{2}}\delta_{2}+D_{x_{3}}\delta_{3}\right)=\left(\Psi -\delta_{1}U_{x_{1}}-\delta_{2}U_{x_{2}}-\delta_{3}U_{x_{3}}-\delta_{4}U_{\tau }\right)\frac{\delta L}{\delta U}\\ & +D_{\tau }G_{1}+D_{x_{1}}G_{2}+D_{x_{2}}G_{3}+D_{x_{3}}G_{4}, \end{array} \end{array}$$ (131)

where,

$$\Lambda^{\left[1\right]}=\Lambda +\Psi^{x_{1}}\frac{\partial }{\partial U_{x_{1}}}+\Psi^{x_{2}}\frac{\partial }{\partial U_{x_{2}}}+\Psi^{x_{3}}\frac{\partial }{\partial U_{x_{3}}}+\Psi^{\tau }\frac{\partial }{\partial U_{\tau }},$$

and $G_{i}\left(x_{1},x_{2}x_{3},\tau ,U\right),i=1,2,3,4$ are gauge terms. We compare the coefficients of derivatives of *U* in the above equation, we obtain the determining system, and then solving this system gives

$$\begin{array}{c} \begin{array}{cc} & A\delta_{2_{x_{2}}}-A\delta_{1_{x_{1}}}+A\delta_{3_{x_{3}}}+A\delta_{4_{\tau }}+2A\Psi_{U}+C\Psi_{x_{1}}=0,\\ & B\delta_{1_{x_{1}}}-B\delta_{2_{x_{2}}}+B\delta_{3_{x_{3}}}+B\delta_{4_{\tau }}+2B\Psi_{U}+D\Psi_{x_{1}}=0,\\ & B\delta_{1_{x_{1}}}+B\delta_{2_{x_{2}}}-B\delta_{3_{x_{3}}}+B\delta_{4_{\tau }}+2B\Psi_{U}+D\Psi_{x_{1}}=0,\\ & 2\Psi_{U}+\delta_{1_{x_{1}}}+\delta_{2_{x_{2}}}+\delta_{3_{x_{3}}}-\delta_{4_{\tau }}+2\gamma \delta_{4}=0,\\ & 3\Psi_{U}-2\delta_{1_{x_{1}}}+\delta_{2_{x_{2}}}+\delta_{3_{x_{3}}}+\delta_{4_{\tau }}=0,\\ & 3\Psi_{U}-\delta_{2_{x_{2}}}+\delta_{3_{x_{3}}}+\delta_{4_{\tau }}=0,\\ & 3\Psi_{U}+\delta_{2_{x_{2}}}-\delta_{3_{x_{3}}}+\delta_{4_{\tau }}=0,\\ & \delta_{1_{x_{2}}}=\delta_{1_{x_{3}}}=\delta_{1_{U}}=0,\\ & \delta_{2_{x_{1}}}=\delta_{2_{U}}=0,\\ & \delta_{2_{x_{3}}}+\delta_{3_{x_{2}}}=0,\\ & \delta_{3_{x_{1}}}=\delta_{3_{U}}=0,\\ & \delta_{4_{x_{1}}}=\delta_{4_{x_{2}}}=\delta_{4_{x_{3}}}=\delta_{4_{U}}=0,\\ & \Psi_{x_{2}}=\Psi_{x_{3}}=G_{U}^{3}=G_{U}^{4}=0,\\ & A\Psi_{x_{1}}-G_{U}^{2}=0,\\ & \gamma \Psi -\Psi_{\tau }-G_{U}^{1}=0,\\ & -\gamma \delta_{1}+\delta_{1_{\tau }}=0,\\ & -\gamma \delta_{2}+\delta_{2_{\tau }}=0,\\ & -\gamma \delta_{3}+\delta_{3_{\tau }}=0,\\ & G_{\tau }^{1}+G_{x_{1}}^{2}+G_{x_{2}}^{3}+G_{x_{3}}^{4}=0. \end{array} \end{array}$$ (132)

The solution of the above system follows

$$\delta_{1}=C_{1}e^{\gamma \tau },\;\delta_{2}=\left(C_{2}x_{3}+C_{3}\right)e^{\gamma \tau },\;\delta_{3}=\left(-C_{2}x_{2}+C_{4}\right)e^{\gamma \tau },\;\delta_{4}=0,\;\Psi =C_{5}+C_{6}e^{\gamma \tau }$$

$$G^{1}=C_{5}\gamma U-X_{x_{2}}+\int \left(-h_{x_{1}}-G_{x_{3}}\right)dt+p\left(x_{1},x_{2},x_{3}\right),\;G^{2}=h\left(x_{1},x_{2},x_{3},\tau \right),$$

$$G^{3}=X_{\tau },\;G^{4}=g\left(x_{1},x_{2},x_{3},\tau \right).$$

By taking *f* = *g* = *h* = *p* = 0,

we get,

$$G^{1}=C_{5}\gamma U,\;\;\;\;G^{2}=G^{3}=G^{4}=0.$$

Finally, we had the following partial Noether symmetry generators

$$\Lambda_{1}=e^{\gamma \tau }\frac{\partial }{\partial x_{1}},\;\Lambda_{2}=e^{\gamma \tau }\frac{\partial }{\partial x_{2}},\;\Lambda_{3}=e^{\gamma \tau }\frac{\partial }{\partial x_{2}},\;\Lambda_{4}=e^{\gamma \tau }\left(x_{3}\frac{\partial }{\partial x_{2}}-x_{2}\frac{\partial }{\partial x_{3}}\right),$$

$$\Lambda_{5}=e^{\gamma \tau }\frac{\partial }{\partial U},\;\Lambda_{6}=\frac{\partial }{\partial U},and\;G^{1}=\gamma U.$$

### 7.3 Conserved vectors

For Λ_1_, we have *δ*_1_ = *e*^*γτ*^, *δ*_2_ = *δ*_3_ = *δ*_4_ = Ψ = 0 and $G_{1}=G_{2}=G_{3}=G_{4}=0$, we follow from Eq (68)

$$T^{\tau }=G_{1}-L\delta_{4}-\left(\Psi -U_{x_{1}}\delta_{1}-U_{x_{2}}\delta_{2}-U_{x_{3}}\delta_{3}-U_{\tau }\delta_{4}\right)\frac{\partial L}{\partial U_{\tau }}\cdot$$

This lead to the component of the conserved vector given by,

$$T^{\tau }=-e^{\gamma t}U_{x_{1}}U_{\tau }$$

$$T^{x_{1}}=G_{2}-L\delta_{1}-\left(\Psi -U_{x_{1}}\delta_{1}-U_{x_{2}}\delta_{2}-U_{x_{3}}\delta_{3}-U_{\tau }\delta_{4}\right)\frac{\partial L}{\partial U_{x_{1}}},$$

we get

$$T^{x_{1}}=e^{\gamma \tau }\left(\frac{A}{2}U_{x_{1}}^{2}+\frac{C}{3}U_{x_{1}}^{3}+\frac{U_{\tau }^{2}}{2}-\frac{B}{2}U_{x_{2}}^{2}-\frac{B}{2}U_{x_{3}}^{2}\right),$$

$$T^{x_{2}}=G_{3}-L\delta_{2}-\left(\Psi -U_{x_{1}}\delta_{1}-U_{x_{2}}\delta_{2}-U_{x_{3}}\delta_{3}-U_{\tau }\delta_{4}\right)\frac{\partial L}{\partial U_{x_{2}}},$$

this implies

$$T^{x_{y}}=e^{\gamma \tau }\left(BU_{x_{1}}U_{x_{2}}+DU_{x_{1}}^{2}U_{x_{2}}\right),$$

$$T^{x_{3}}=G_{4}-L\delta_{3}-\left(\Psi -U_{x_{1}}\delta_{1}-U_{x_{2}}\delta_{2}-U_{x_{3}}\delta_{3}-U_{\tau }\delta_{4}\right)\frac{\partial L}{\partial U_{x_{3}}},$$

we obtain the result

$$T^{x_{3}}=e^{\gamma \tau }\left(BU_{x_{1}}U_{x_{3}}+DU_{x_{1}}^{2}U_{x_{3}}\right).$$

For Λ_2_, *δ*_2_ = *e*^*γτ*^, *δ*_1_ = *δ*_3_ = *δ*_4_ = Ψ = 0 and $G_{1}=G_{2}=G_{3}=G_{4}=0$. From Eq (68), the conserved vector associated with Λ_2_ becomes

$$\begin{array}{c} \begin{array}{cc} & T^{\tau }=-e^{\gamma \tau }U_{x_{2}}U_{\tau },\\ & T^{x_{1}}=e^{\gamma \tau }\left(AU_{x_{1}}U_{x_{2}}+\frac{C}{2}U_{x_{1}}^{2}U_{x_{2}}+\frac{D}{2}\left(U_{x_{2}}^{3}+U_{x_{2}}U_{x_{3}}^{2}\right)\right),\\ & T^{x_{2}}=e^{\gamma \tau }\left(-\frac{A}{2}U_{x_{1}}^{2}-\frac{C}{6}U_{x_{1}}^{3}+\frac{U_{\tau }^{2}}{2}+\frac{B}{2}\left(U_{x_{2}}^{2}-U_{x_{3}}^{2}\right)+\frac{D}{2}\left(U_{x_{1}}U_{x_{2}}^{2}-U_{x_{1}}U_{x_{3}}^{2}\right)\right),\\ & T^{x_{3}}=e^{\gamma \tau }U_{x_{2}}\left(BU_{x_{3}}+DU_{x_{1}}U_{x_{3}}\right). \end{array} \end{array}$$ (133)

For Λ_3_, *δ*_3_ = *e*^*γτ*^, *δ*_1_ = *δ*_2_ = *δ*_4_ = Ψ = 0 and $G_{1}=G_{2}=G_{3}=G_{4}=0$. From Eq (68), the conserved vector associated with Λ_3_ becomes

$$\begin{array}{c} \begin{array}{cc} & T^{\tau }=-e^{\gamma \tau }U_{x_{3}}U_{\tau },\\ & T^{x_{1}}=e^{\gamma \tau }\left(AU_{x_{1}}U_{x_{3}}+\frac{C}{2}U_{x_{1}}^{2}U_{x_{3}}+\frac{D}{2}\left(U_{x_{2}}^{2}U_{x_{3}}+U_{x_{3}}^{3}\right)\right),\\ & T^{x_{2}}=e^{\gamma \tau }U_{x_{2}}\left(BU_{x_{3}}+DU_{x_{1}}U_{x_{3}}\right),\\ & T^{x_{3}}=e^{\gamma \tau }\left(-\frac{A}{2}U_{x_{1}}^{2}-\frac{C}{6}U_{x_{1}}^{3}+\frac{U_{\tau }^{2}}{2}+\frac{B}{2}\left(-U_{x_{2}}^{2}+U_{x_{3}}^{2}\right)+\frac{D}{2}\left(-U_{x_{1}}U_{x_{2}}^{2}+U_{x_{1}}U_{x_{3}}^{2}\right)\right). \end{array} \end{array}$$ (134)

For Λ_4_, we have *δ*_2_ = *x*_3_*e*^*γτ*^, *δ*_3_ = −*x*_2_*e*^*γτ*^, *δ*_1_ = *δ*_4_ = Ψ = 0 and $G_{1}=G_{2}=G_{3}=G_{4}=0$. From Eq (68), the conserved vector associated with Λ_4_ becomes

$$\begin{array}{c} \begin{array}{cc} & T^{\tau }=\left(-x_{3}U_{x_{2}}+x_{2}U_{x_{3}}\right)e^{\gamma \tau }U_{\tau },\\ & T^{x_{1}}=e^{\gamma \tau }\left(zU_{x_{2}}-x_{2}U_{x_{3}}\right)\left(AU_{x_{1}}+\frac{C}{2}U_{x_{1}}^{2}+\frac{D}{2}\left(U_{x_{2}}^{2}+U_{x_{3}}^{2}\right)\right),\\ & T^{x_{2}}=-x_{3}e^{\gamma \tau }\left(\frac{A}{2}U_{x_{1}}^{2}+\frac{C}{6}U_{x_{1}}^{3}-\frac{U_{\tau }^{2}}{2}-\frac{B}{2}\left(U_{x_{2}}^{2}-U_{x_{3}}^{2}\right)-\frac{D}{2}\left(U_{x_{1}}U_{x_{2}}^{2}-U_{x_{1}}U_{x_{3}}^{2}\right)\right)-yU_{x_{2}}U_{x_{3}}e^{\gamma \tau }\left(B+DU_{x_{1}}\right),\\ & T^{x_{3}}=x_{2}e^{\gamma \tau }\left(\frac{A}{2}U_{x_{1}}^{2}+\frac{C}{6}U_{x_{1}}^{3}-\frac{U_{\tau }^{2}}{2}+\frac{B}{2}\left(U_{x_{2}}^{2}-U_{x_{3}}^{2}\right)+\frac{D}{2}\left(U_{x_{1}}U_{x_{2}}^{2}-U_{x_{1}}U_{x_{3}}^{2}\right)\right)+zU_{x_{2}}U_{x_{3}}e^{\gamma \tau }\left(B+DU_{x_{1}}\right). \end{array} \end{array}$$ (135)

For Λ_5_, Ψ = *e*^*γτ*^, *δ*_1_ = *δ*_2_ = *δ*_3_ = *δ*_4_ = 0 and $G_{1}=G_{2}=G_{3}=G_{4}=0$. From Eq (68), the conserved vector associated with Λ_5_ becomes

$$\begin{array}{c} \begin{array}{cc} & T^{\tau }=e^{\gamma \tau }U_{\tau },\\ & T^{x_{1}}=e^{\gamma \tau }\left(-AU_{x_{1}}-\frac{C}{2}U_{x_{1}}^{2}-\frac{D}{2}\left(U_{x_{2}}^{2}+U_{x_{3}}^{2}\right)\right),\\ & T^{x_{2}}=-e^{\gamma \tau }\left(BU_{x_{2}}+DU_{x_{1}}U_{x_{2}}\right),\\ & T^{x_{3}}=-e^{\gamma \tau }\left(BU_{x_{3}}+DU_{x_{1}}U_{x_{3}}\right). \end{array} \end{array}$$ (136)

For Λ_6_, we have Ψ = 1, *δ*_1_ = *δ*_2_ = *δ*_3_ = *δ*_4_ = 0 and $G_{1}=\gamma U,G_{2}=G_{3}=G_{4}=0$. From Eq (68), the conserved vector associated with Λ_6_ becomes

$$\begin{array}{c} \begin{array}{cc} & T^{\tau }=\gamma U+U_{\tau },\\ & T^{x_{1}}=-\left(AU_{x_{1}}+\frac{C}{2}U_{x_{1}}^{2}+\frac{D}{2}\left(U_{x_{2}}^{2}+U_{x_{3}}^{2}\right)\right),\\ & T^{x_{2}}=-\left(BU_{x_{2}}+DU_{x_{1}}U_{x_{2}}\right),\\ & T^{x_{3}}=-\left(BU_{x_{3}}+DU_{x_{1}}U_{x_{3}}\right). \end{array} \end{array}$$ (137)

## 8 Graphical analysis

**Fig 1:**
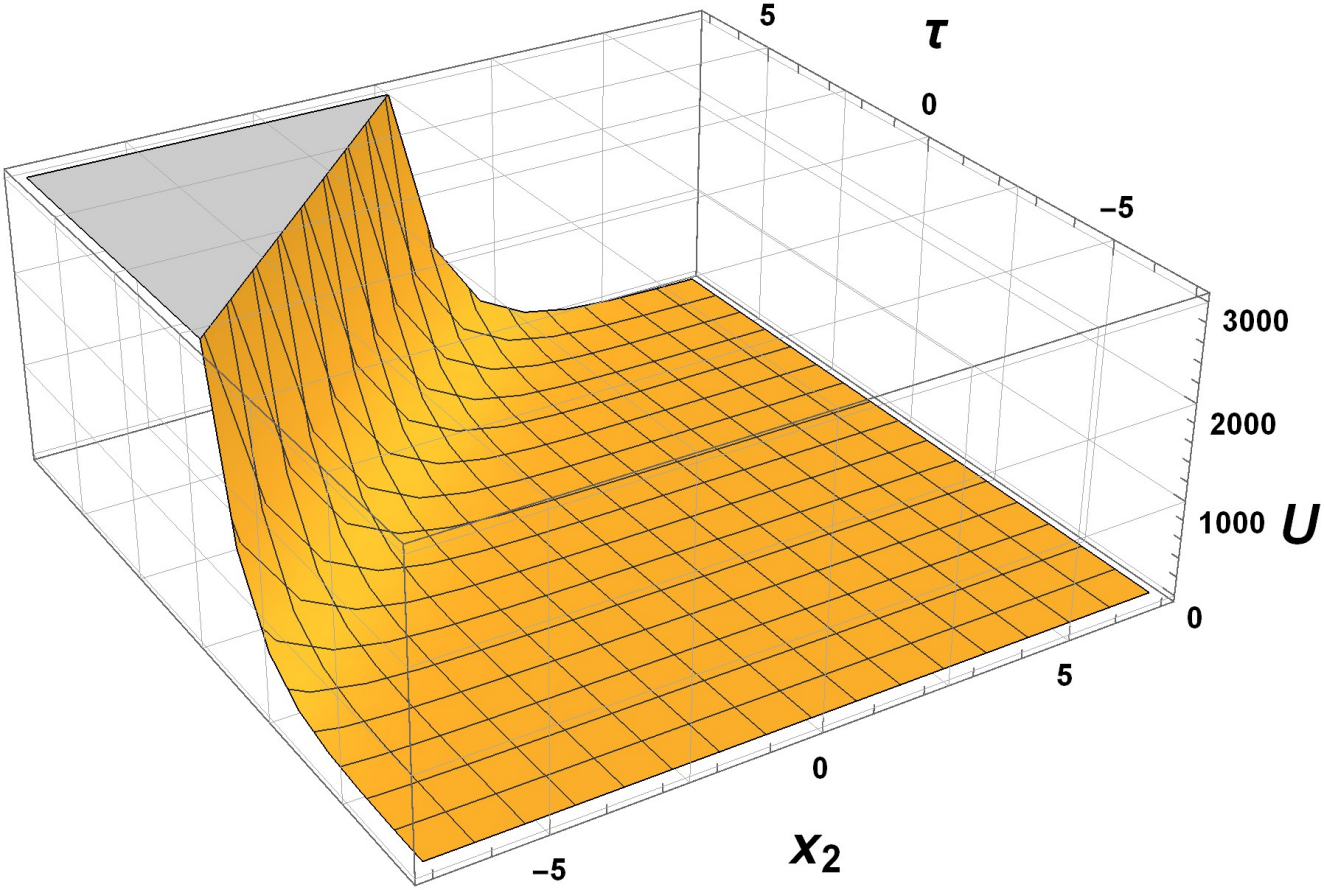
Behaviour of three-dimensional elastic motion by (100) with parameters *c*_1_ = 1, *c*_2_ = 3, *B* = 2 and *γ* = 1.

**Fig 2:**
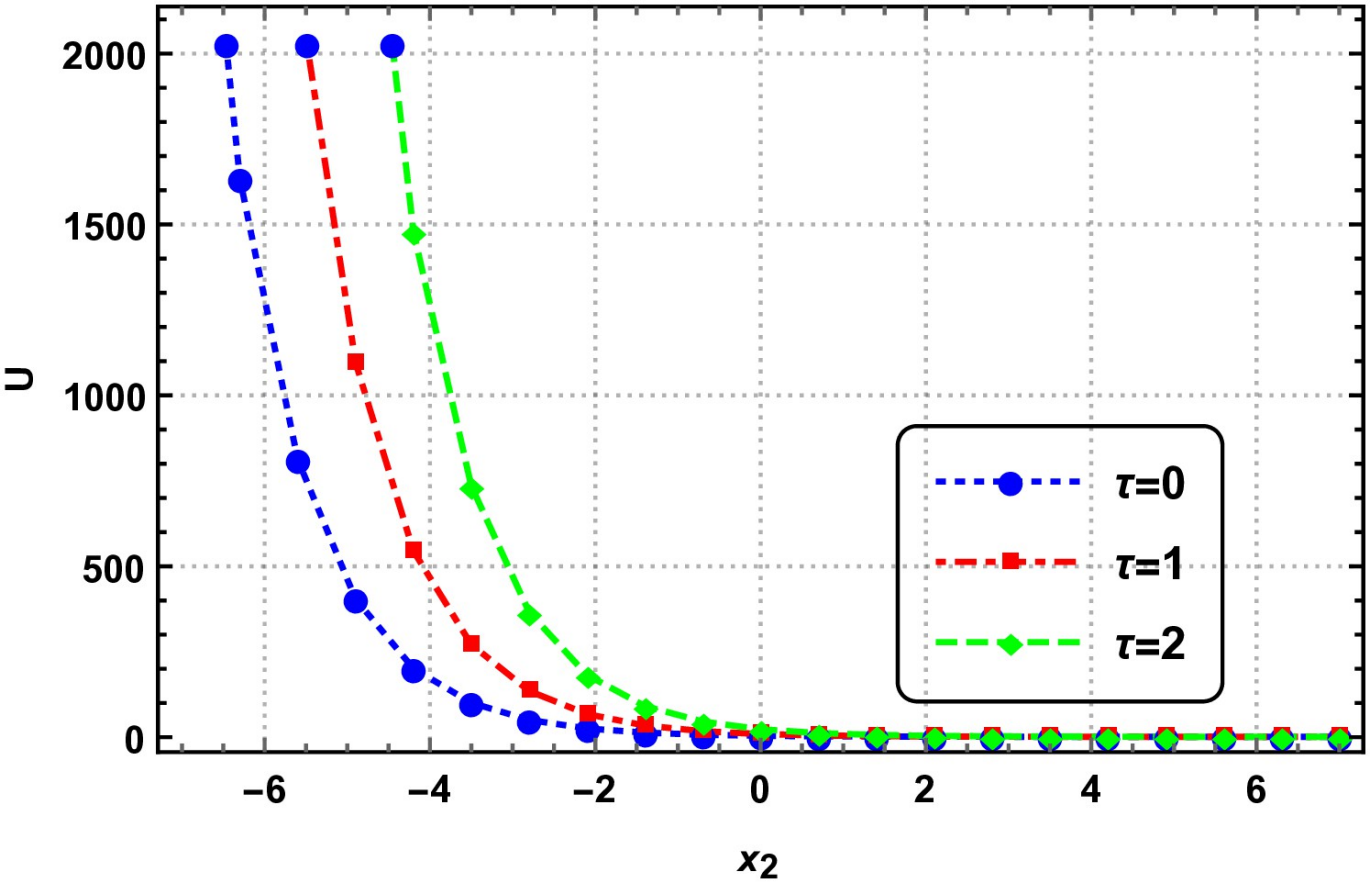
Behaviour of two-dimensional elastic motion by (100) with parameters *c*_1_ = 1, *c*_2_ = 3, *B* = 2 and *γ* = 1.

**Fig 3:**
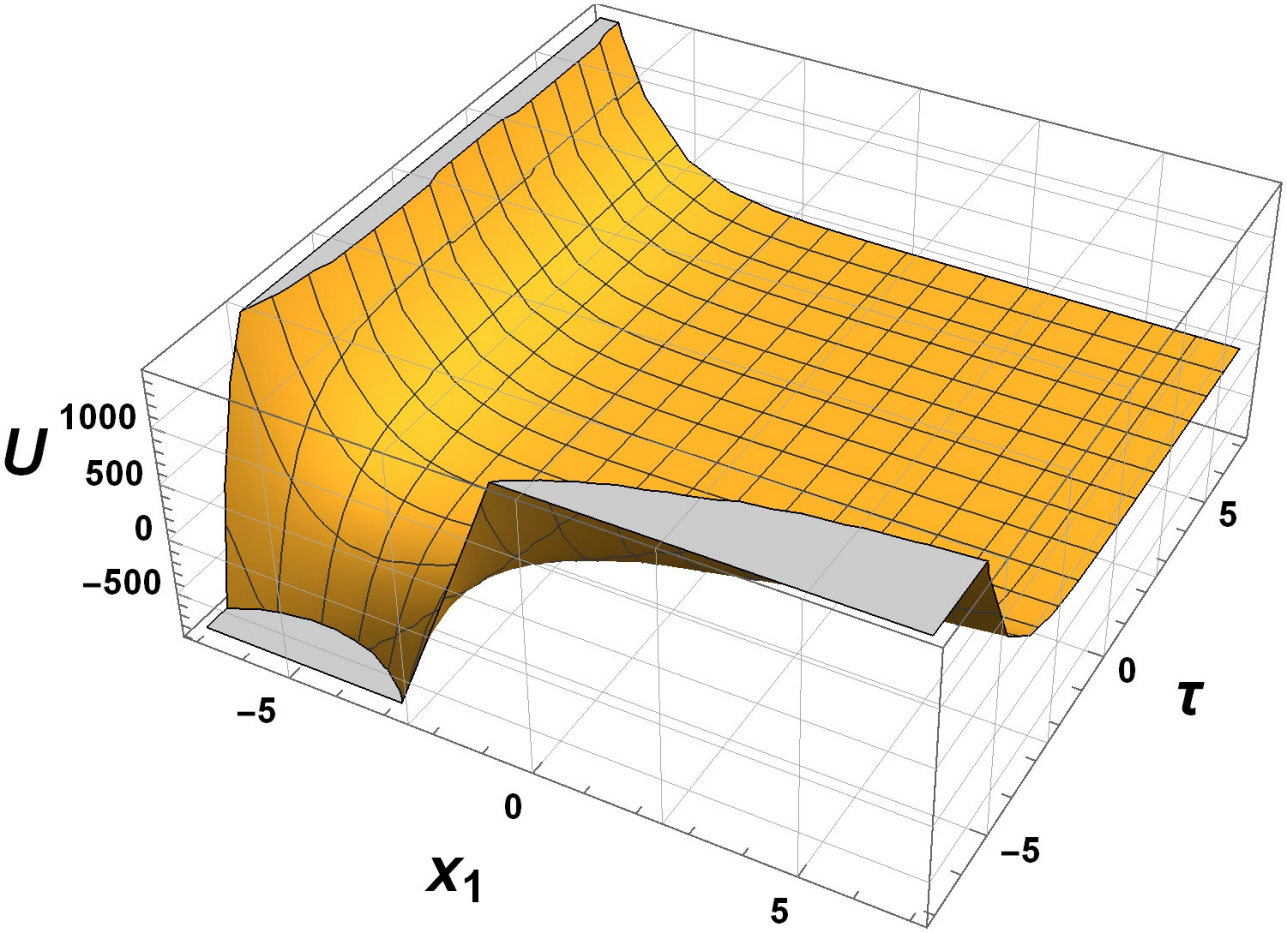
Behaviour of three-dimensional elastic motion by (109) with parameters *c*_1_ = 1, *c*_2_ = 2 and *γ* = 1.

**Fig 4:**
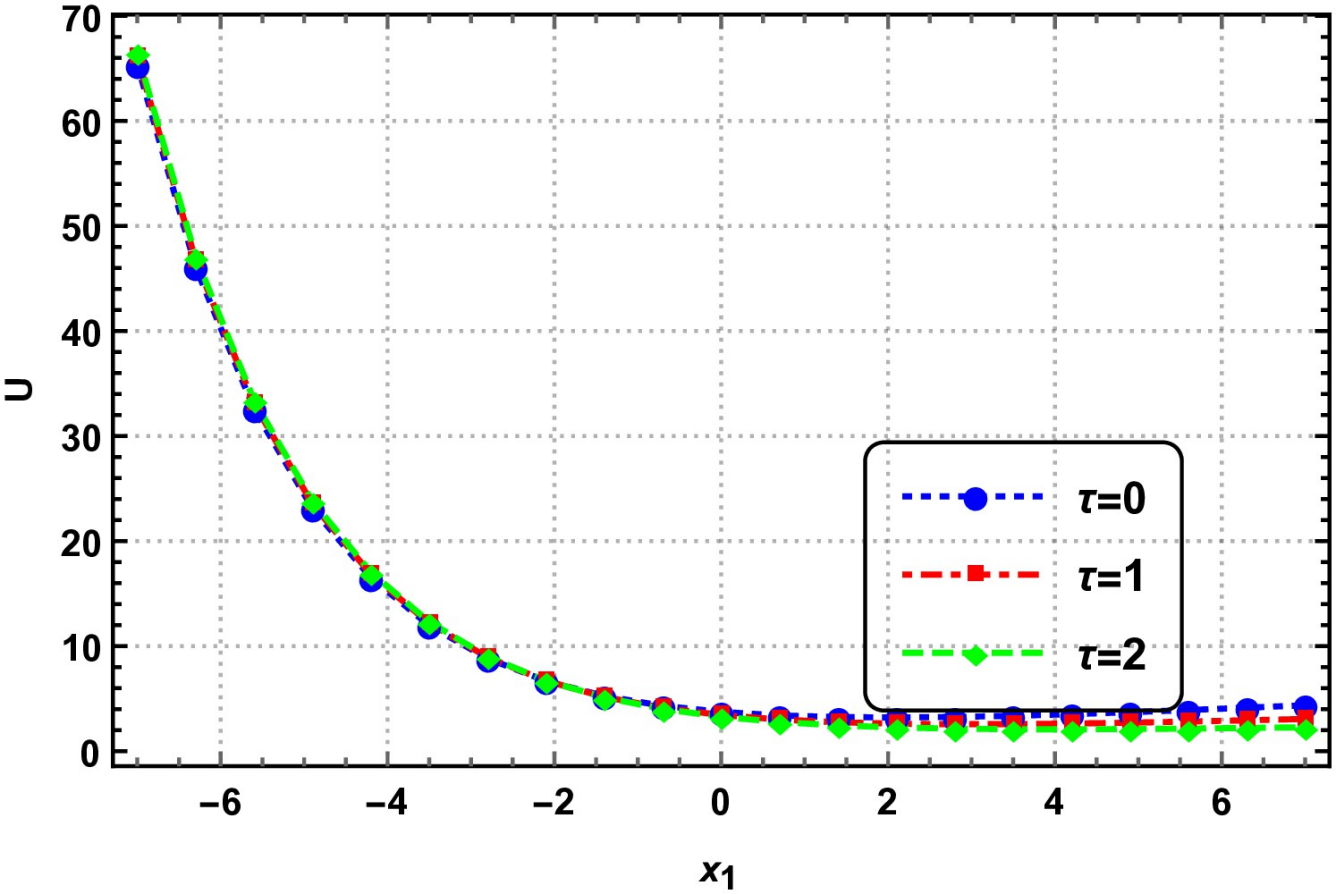
Behaviour of two-dimensional elastic motion by (109) with parameters *c*_1_ = 1, *c*_2_ = 2 and *γ* = 1.

In this section, we explore the graphical analysis of the nonlinear elastic wave equations, which proves crucial when dealing with exact solutions. This analysis helps us grasp the intricate nature of complex physical phenomena. Figures labeled as Figs 1–4 exemplify the interplay of two and three-dimensional motion of elastic waves. Notably, Figs 1 and 2 validate the presence of exponential within the elastic wave equations. These figures are based on specific parameter values, providing valuable insights into the behavior of the elastic waves.

## 9 Conclusions

In this study, our main objective was to investigate the (3+1)-dimensional elasticity wave equations using the widely employed Lie group method. This research is novel because no previous studies have applied the Lie group method to these equations. Our exploration provides unique analytical solutions that are invariant under certain transformations or symmetry generators, highlighting the novelty of our results. Additionally, we derived the conservation laws of linear momentum and energy within the model. To achieve this, we formulated classical and partial Lagrangians for the elasticity wave equation and its damped version, respectively. This exploration of conservation laws using Noether’s theorem is presented for the first time in the literature for the (3+1)-dimensional elasticity wave equations, as is the application of the partial Lagrangian approach to these models. Our results demonstrate the effectiveness of the partial Lagrangian approach when a classical Lagrangian is unavailable, extending the applications of variational principles.

We accomplished this goal by thoroughly discussing both Noether’s approach and partial Noether’s approach to elasticity equations, resulting in the successful derivation of conserved vectors. The soundness of the Lie group method was confirmed through our analysis. Furthermore, we found that our adopted technique was also suitable for exploring group invariant solutions. The promising results have motivated us to further research and study additional classes within the elasticity medium. These findings pave the way for future investigations in the field of elasticity equations.
